# Supplementary material for: Infant formula composition and educational performance: a protocol to extend follow-up for a set of randomised controlled trials using linked administrative education records
Source: BMJ Open. 2020 Jul 23;10(7):e035968. doi: 10.1136/bmjopen-2019-035968 (PMC7380883; doi:10.1136/bmjopen-2019-035968)
Supplement: Supplementary data [file bmjopen-2019-035968supp001.pdf]

bmjopen-2019-035968 R.2 Supplementary material

Supplementary Table 1 Trial Characteristics

	**RCT 1**	**RCT 2**	**RCT 3**	**RCT 4**	**RCT 5**	**RCT 6**	**RCT 7**
Intervention	Nutrient-enriched	Nutrient-enriched	LCPUFA	LCPUFA	Nucleotides	Iron	Sn-2 palmitate
Population	Preterm	SGA Term	Preterm	Term	Term	Term	Term
Years randomised	1993-96	1993-96	1993-96	1993-95	2000-02	1993-94	1995-96
Eligibility	<1750g bw <37w ga	bw <10th centile ≥37w ga	≤1750g bw ≤37w ga	≥37w ga	≥37w ga	bw > 2500g, ≥37w ga age: 9m	≥37w ga, bw>5th centile
Control formula:	Term formula	Term formula	Preterm formula	Term formula	Term formula	Term formula or cow's milk	Term formula
Intervention: Control formula with added:	Macro nutrients and vitamins	Macro nutrients and vitamins	DHA and AA	DHA and AA	Nucleotides	12 mg/L iron	Sn-2 palmitate
Timing of intervention	1w < discharge - 9m	Birth – 12w	Birth – 3w	Birth – 6m	Birth – 20w	9m - 18m	Birth – 12w
Centre(s)	Nottingham Leicester Cambridge Ipswich	Nottingham Leicester Cambridge	Nottingham Leicester	Nottingham Leicester	Nottingham Leicester	Nottingham Leicester Norwich	Cambridge
Total N	296	475	284	447	296	492	323
Intervention	113	152	96	155	99	161	103
Control (1)	115	147	100	154	97	165	100
Control (2)	-	-	-	-	-	166	-
Non-randomised breastfed reference	68	176	88	138	100	-	120
*% Response at last cognitive follow-up (test, age) **	84% (Bayley score, 18m)	6% (IQ score, 17 years)	9% (IQ score, 16 years)	55% (IQ score, 5.5 years)	81% (Head circumference, 20 weeks)	36% (Bayley score, 18m)	No cognitive follow-up
References	[1]	[2,3]	[4,5]	[6,7]	[8,9]	[10,11]	[12,13]

bw=birthweight, ga=gestational age, d=days, w=week(s), m=moths, LCPUFA=Long Chain Polyunsaturated Fatty Acids, SGA=small-for-gestational-age, m=months, DHA= docosahexaenoic acid, AA= arachidonic acid *includes unpublished follow-ups

bmjopen-2019-035968 R.2 Supplementary material

Data cleaning methods:

The party who conducts the linkage (the Fischer Family Trust, FFT) provides up to three likely matching pupil IDs and their school records per participant. Only one of these records can be a true match and it is possible that none of the linked pupil IDs is a true match, as participants might have emigrated, died or attended private schools which do not transmit data to the national pupil database. We will check and cross validate the data provided by FFT extensively, using auxiliary variables from the trials (such as known death) and linkage confidence flags supplied by FFT (Supplementary Table 2).

Supplementary Table 2 Possible match levels

Identifier	Description
First name	First name and other first name both exact match
	First name matches other name in both directions
	First name exact match
	First name matches other name
	Other name exact match
	First name truncated at any hyphen matches
	First name matches via common name alternatives lookup
	Pattern match function
	Pattern match function - AND first character of first name matches
	First name / surname match in both directions
	No Link
Surname	Surname exact match (including alternative surnames)
	Surname truncated at any hyphen matches
	Pattern match function
	Pattern match function - AND first character of surname matches
	First name / surname match in both directions
	No Link
Date of Birth	DOB exact match
	Day on source matches month on match, and vice versa; year matches (i.e. transposed date)
	Day and month match (i.e. wrong year)
	Day and year match (i.e. wrong month)
	Month and year match (i.e. wrong day)
	Either source or match DOB is 1st January; year matches
	Either source or match DOB is 1st September; year matches
	No Link
Location	Postcode exact match
	Local Authority match
	Neighbouring / nearby Local Authority match

Verfürden M, *et al. BMJ Open* 2020; 10:e035968. doi: 10.1136/bmjopen-2019-035968

bmjopen-2019-035968 R.2 Supplementary material

No Link

We will calculate match-weights for each participant-pupil pair according to a probabilistic method adapted from Fellegi and Sunter.[14] These match-weights will then be used to rank the pairs according to their probability of being a true match. For each match category (Supplementary Table 2) we will derive proxy-m and proxy-u probabilities which will then be used to produce the match weight.

M-probability: probability of agreeing on a category given that it is a true match
U-probability: probability of agreeing on a category given by chance

The original method requires a set of participant-pupil pairs that are certain true matches and a set of participant-pupil pairs that are certain false matches. Due to the de-identified nature of the data, such information will not be available and thus needs to be approximated. True matches will be defined as participants perfectly matching all categories (except the one we are calculating the probability for). Certain false matches will be defined as participants disagreeing in all categories (except the one we are calculating the probability for). This will yield a total of eight reference datasets. For each linkage flag (sex, first name, last name, date of birth, location) we will then calculate the variable weight using:

$$\log 2\ (m/u)$$

The final match weight for a participant-pupil pair will be calculated using the sum of all weights in a pair:

$$\sum \log 2\ (m/u)$$

At this stage each participant will still have up to three multiple possible matches.
First, we will drop all participants that linked but where their age at first link was >17 and they do not agree with the record on date of birth. This is because the date of birth in the trial data is very likely to be correct as it corresponds to the recruitment periods and was confirmed repeatedly during the follow-up period. We will also discard all records for children that died before school age (4 years) where information on death and time of death during the trial period is available.

We will then rank participant-pupil pairs according to their match weights. If the highest match-weight for a participant is 10% above that of the second best match, we will automatically keep the best match and discard the other(s). If the difference is lower, we will manually review these

Verfürden M, *et al. BMJ Open* 2020; 10:e035968. doi: 10.1136/bmjopen-2019-035968

records to decide which one is the best match (if any). Decisions taken at manual review will be reported to ensure reproducibility. We will then choose two cut-offs based on visual inspection of the match probabilities; one threshold to maximise sensitivity (many true positive links) and one to maximise specificity (few false positive links). To determine how representative each sample defined by these cut-offs is of the original randomised cohort, we will compare them across the variables that were measured at randomisation. We will use the bigger, more sensitive sample in our analyses but use the smaller, more specific sample in sensitivity analyses.

bmjopen-2019-035968 R.2 Supplementary material

Trial formula compositions:

RCT 1: Preterm nutrient enriched formula (post-discharge) vs standard formula

per 100 ml	Standard formula	High-energy formula
Energy (kcal)	68	72
Protein (g)	1.45	1.85
Casein	0.56	0.72
Whey	0.89	1.13
Carbohydrate (g)	6.96	7.24
Lactose (g)	6.96	6.20
Maltodextrin (g)	-	1.04
Fat (g)	3.82	3.96
Minerals		
Calcium (mg)	39	70
Phosphorus (mg)	27	35
Sodium (mg)	17	22
Chloride (mg)	45	45
Potassium (mg)	57	78
Iron (mg)	0.65	0.65
Zinc (mg)	0.34	0.60
Copper (µg)	42	57
Iodine (µg)	4.5	4.5
Magnesium (mg)	5.2	5.2
Manganese (µg)	3.4	5
Vitamins		
Vitamin A (µg)	100	100
Thiamine B_1 (mg)	42	95
Riboflavin B_2 (mg)	55	100
Niacin B_3 (µg)		
Pantothenic acid B_5 (mg)	0.23	0.40
Pyridoxine B_6 (µg)	35	80
Biotin B_7 (µg)	1.0	1.1
Inositol B_8 (mg)		
Folate B_9 (µg)	3.4	25
Cyanocobalamin B_{12} (µg)	0.14	0.2
Vitamin C (mg)	6.9	15
Vitamin D (µg)	1.0	1.3
Vitamin E (mg)	0.48	1.5
Vitamin K (µg)	2.7	6.0
Choline (mg)	4.8	5.1
Taurine (mg)	5	5.1
Carnitine (mg)	-	1.1

Verfürden M, *et al. BMJ Open* 2020; 10:e035968. doi: 10.1136/bmjopen-2019-035968

bmjopen-2019-035968 R.2 Supplementary material

RCT 2: Term SGA nutrient enriched formula vs standard formula

per 100 ml	Standard formula	High-energy formula
Energy (kcal)	68	72
Protein (g)	1.45	1.85
Casein	0.56	0.72
Whey	0.89	1.13
Carbohydrate (g)	6.96	7.24
Lactose (g)	6.96	6.20
Maltodextrin (g)	-	1.04
Fat (g)	3.82	3.96
Minerals		
Calcium (mg)	39	70
Phosphorus (mg)	27	35
Sodium (mg)	17	22
Chloride (mg)	45	45
Potassium (mg)	57	78
Iron (mg)	0.65	0.65
Zinc (mg)	0.34	0.60
Copper (µg)	42	57
Iodine (µg)	4.5	4.5
Magnesium (mg)	5.2	5.2
Manganese (µg)	3.4	5
Vitamins		
Vitamin A (µg)	100	100
Thiamine B_1 (mg)	42	95
Riboflavin B_2 (mg)	55	100
Niacin B_3 (µg)		
Pantothenic acid B_5 (mg)	0.23	0.40
Pyridoxine B_6 (µg)	35	80
Biotin B_7 (µg)	1.0	1.1
Inositol B_8 (mg)		
Folate B_9 (µg)	3.4	25
Cyanocobalamin B_{12} (µg)	0.14	0.2
Vitamin C (mg)	6.9	15
Vitamin D (µg)	1.0	1.3
Vitamin E (mg)	0.48	1.5
Vitamin K (µg)	2.7	6.0
Choline (mg)	4.8	5.1
Taurine (mg)	5.0	5.1
Carnitine (mg)	-	1.1

RCT 3: Preterm LCPUFA-enriched formula vs standard preterm formula

per 100 ml	Standard formula	LCPUFA-enriched formula
Energy (kcal)	70	70

Verfürden M, *et al. BMJ Open* 2020; 10:e035968. doi: 10.1136/bmjopen-2019-035968

bmjopen-2019-035968 R.2 Supplementary material

Protein (g)	2.0	2.0
Casein	0.8	0.8
Whey	1.2	1.2
Carbohydrate (g)	7.7	7.7
Total fat (g)	3.5	3.5
Fatty acid composition (g/ 100 g fat)		
C8:0 caprylic	0.7	0.6
C10:0 capric	1.2	1.1
C12:0 lauric	6.3	4.9
C14:0 myristic	5.6	5.6
C16:0 palmitic	25.8	26.3
C18:0 stearic	8.2	8.5
C18:1 oleic	32.6	32.9
C18:2 n-6 linoleic	10.6	12.0
C18:3 n-6 γ-linolenic	0.1	0.4
C18:3 n-6 α-linolenic	0.7	0.6
C20:0 arachidic	0.4	0.3
C20:1 n-9 eicosanoic	0.2	0.3
C20:4 n-6 AA	-	0.31
C20:5 n-3 eicosapentaenoic	-	0.04
C22:0	0.3	0.2
C22:6 n-3 DHA	-	0.17
C24:0	0.2	0.1
Other fatty acids	7.1	4.62
Cholesterol	-	7.73
Minerals		
Calcium (mg)	70	70
Phosphorus (mg)	35	42
Sodium (mg)	30	27
Potassium (mg)	75	71
Iron (mg)	0.1	0.07
Zinc (mg)	0.39	0.4
Vitamins		
Vitamin A (µg)	63	63
Vitamin D (µg)	2.1	2.1
Vitamin E (mg)	2.0	2.0
Vitamin K (µg)	2.8	3.0

RCT 4: Term LCPUFA-enriched formula vs standard term formula

per 100 ml	Standard formula	LCPUFA-enriched formula
Energy (kcal)	67	67
Protein (g) (casein:whey 40:60)	1.5	1.5
Carbohydrate (g)	7.6	7.7
Lactose (g)	7.6	7.7
Total fat (g)	3.4	3.4
Fatty acid composition (% total fat)		
C8:0 caprylic	0.8	2.0
C10:0 capric	2.4	2.0

Verfürden M, *et al. BMJ Open* 2020; 10:e035968. doi: 10.1136/bmjopen-2019-035968

C12:0 lauric	1.6	12.3
C14:0 myristic	8.6	5.2
C16:0 palmitic	23.3	25.6
C18:0 stearic	10.2	4.6
C18:1 oleic	32.7	29.7
C18:2 n-6 linoleic	12.4	15.9
C18:3 n-6 α-linolenic	1.1	1.4
C20:4 n-6 AA	-	0.30
C20:5 n-3 eicosapentaenoic	-	0.01
C22:6 n-3 DHA	-	0.32
Cholesterol	<0.5	0.8
Minerals		
Iron (mg)	0.8	0.8

RCT 5: Nucleotide-enriched formula vs standard formula

per 100 ml	Standard formula	Nucleotide-enriched formula
Energy (kcal)	68	68
Protein (g) (casein:whey 40:60)	1.5	1.5
Casein	0.6	0.6
Whey	0.9	0.9
Carbohydrate as lactose (g)	7.0	7.0
Total fat (g)	3.8	3.8
C18:2 n-6 linoleic (mg)	350	350
C18:3 n-6 γ-linolenic (mg)	33	33
C18:3 n-6 α-linolenic (mg)	44	44
LCPUFAs (mg)	26	26
Nucleotides		
Cytidine monophosphate (mg)	0.3	1.5
Uridine monophosphate (mg)	-	0.5
Adenosine monophosphate (mg)	-	0.6
Guanosine monophosphate (mg)	-	0.2
Inosine monophosphate (mg)	-	0.3
Minerals		
Calcium (mg)	39	39
Magnesium (mg)	5.2	5.2
Manganese (µg)	3.4	3.4
Phosphorus (mg)	27	27
Sodium (mg)	17	17
Potassium (mg)	0.3	0.3
Iron (mg)	0.6	0.6
Zinc (mg)	0.3	0.3
Copper (µg)	42	42
Iodine (µg)	4.5	4.5
Vitamins		
Vitamin A (µg)	100	100
Thiamine B_1 (mg)	42	42
Riboflavin B_2 (mg)	55	55

bmjopen-2019-035968 R.2 Supplementary material

Niacin B_3 (mg)	0.7	0.7
Pantothenic acid B_5 (µg)	0.2	0.2
Biotin B_7 (µg)	1.0	1.0
Vitamin B_8 (µg)	35	35
Folic Acid B_9 (µg)	3.4	3.4
Cyanocobalamin B_{12} (µg)	0.14	0.14
Vitamin C (mg)	6.9	6.9
Vitamin D (µg)	1.0	1.0
Vitamin E (mg)	0.5	0.5
Vitamin K (µg)	2.7	2.7
Choline (mg)	4.8	4.8
Taurine (mg)	5.0	5.0

RCT 6: Iron-enriched formula vs standard formula

per 100 ml	Standard formula	Iron-enriched formula
Energy (kcal)	65	65
Protein (g)	2.5	2.5
Carbohydrate (g)	8.0	8.0
Lactose (g)	6.4	6.4
Maltodextrin	1.6	1.6
Fat (g)	2.8	2.8
Saturated (%)	42.9	42.9
Unsaturated (%)	57.1	57.1
Minerals		
Calcium (mg)	100	100
Phosphorus (mg)	65	65
Magnesium (mg)	7.5	7.5
Sodium (mg)	30	30
Potassium (mg)	100	100
Chloride (mg)	70	70
Iron (mg)	0.9	1.2
Zinc (mg)	0.5	0.5
Iodine (µg)	6.9	6.9
Vitamins		
Vitamin A (µg)	6.9	6.9
Thiamine B_1 (mg)	0.8	0.8
Riboflavin B_2 (mg)	0.12	0.12
Niacin B_3 (µg)	0.61	0.61
Pantothenic acid B_5 (µg)	0.24	0.24
Pyridoxine B_6 (µg)	48	48
Biotin B_7 (µg)	1.71	1.71
Inositol B_8 (mg)		
Folate B_9 (µg)	6.0	6.0
Cyanocobalamin B_{12} (µg)	0.12	0.12

bmjopen-2019-035968 R.2 Supplementary material

Vitamin C (µg)	6.6	6.6
Vitamin D (µg)	1.2	1.2
Vitamin E (mg)	0.75	0.75
Vitamin K (µg)	6.6	6.6

RCT 7: sn-2 Palmitate enriched formula vs standard formula

per 100 ml	Standard formula	sn-2 Palmitate enriched formula
Energy (kcal)	70	73
Protein (g)	1.6	1.6
Carbohydrate (g)	7.1	7.1
Lactose (g)	7.1	7.1
Total fat (g)	3.9	4.2
Fatty acid composition (% total fat)		
C8:0 caprylic	1.6	1.5
C10:0 capric	0.5	0.4
C12:0 lauric	10.3	12.4
C14:0 myristic	4.9	4.7
C16:0 palmitic	19.6 (12% in sn-2)	20.1 (50% in sn-2)
C18:0 stearic	3.9	3.1
C18:1 n-9 oleic	41.3	42
C18:2 n-6 linoleic	12.1	13
C18:3 n-3 α-linolenic	1.9	1.6
Other fatty acids	4.0	1.2
Minerals		
Calcium (mg)	54	57
Phosphorus (mg)	32	33
Sodium (mg)	23	23
Potassium (mg)	76	74
Chloride (mg)	45	44
Magnesium (mg)	6.3	6.3
Manganese (µg)	5.1	5.2
Iron (mg)	0.51	0.57
Zinc (mg)	0.45	0.49
Copper (µg)	44	45
Iodine (µg)	10	10
Vitamins		
Vitamin A (µg)	92	95
Thiamine B_1 (µg)	40	40
Riboflavin B_2 (µg)	100	100
Pyridoxine B_6 (µg)	40	40
Vitamin D (µg)	1.1	1.1
Vitamin K (µg)	5	5
Vitamin E (mg)	1.1	1.1

10

bmjopen-2019-035968 R.2 Supplementary material

References:

1. Lucas A, Fewtrell MS, Morley R, et al. Randomized trial of nutrient-enriched formula versus standard formula for postdischarge preterm infants. Pediatrics 2001;108(3):703-11. [published Online First: 2001/09/05]
2. Fewtrell MS, Morley R, Abbott RA, et al. Catch-up growth in small-for-gestational-age term infants: a randomized trial. The American journal of clinical nutrition 2001;74(4):516-23. doi: 10.1093/ajcn/74.4.516 [published Online First: 2001/09/22]
3. Morley R, Fewtrell MS, Abbott RA, et al. Neurodevelopment in children born small for gestational age: a randomized trial of nutrient-enriched versus standard formula and comparison with a reference breastfed group. Pediatrics 2004;113(3 Pt 1):515-21. [published Online First: 2004/03/03]
4. Fewtrell MS, Morley R, Abbott RA, et al. Double-Blind, Randomized Trial of Long-Chain Polyunsaturated Fatty Acid Supplementation in Formula Fed to Preterm Infants. Pediatrics 2002;110(1):73-82. doi: 10.1542/peds.110.1.73
5. Fewtrell MS, Abbott RA, Kennedy K, et al. Randomized, double-blind trial of long-chain polyunsaturated fatty acid supplementation with fish oil and borage oil in preterm infants. The Journal of pediatrics 2004;144(4):471-9. doi: 10.1016/j.jpeds.2004.01.034 [published Online First: 2004/04/08]
6. Lucas A, Stafford M, Morley R, et al. Efficacy and safety of long-chain polyunsaturated fatty acid supplementation of infant-formula milk: a randomised trial. Lancet (London, England) 1999;354(9194):1948-54. doi: 10.1016/s0140-6736(99)02314-4 [published Online First: 2000/01/06]
7. Singhal A, Morley R, Cole TJ, et al. Infant nutrition and stereoacuity at age 4-6 y. The American journal of clinical nutrition 2007;85(1):152-9. doi: 10.1093/ajcn/85.1.152 [published Online First: 2007/01/09]
8. Singhal A, Kennedy K, Lanigan J, et al. Dietary Nucleotides and Early Growth in Formula-Fed Infants: A Randomized Controlled Trial. Pediatrics 2010;126(4):e946-e53. doi: 10.1542/peds.2009-2609
9. Singhal A, Macfarlane G, Macfarlane S, et al. Dietary nucleotides and fecal microbiota in formula-fed infants: a randomized controlled trial. The American journal of clinical nutrition 2008;87(6):1785-92. [published Online First: 2008/06/11]
10. Morley R, Abbott R, Fairweather-Tait S, et al. Iron fortified follow on formula from 9 to 18 months improves iron status but not development or growth: a randomised trial. Archives of Disease in Childhood 1999;81(3):247-52. doi: 10.1136/adc.81.3.247
11. Singhal A, Morley R, Abbott R, et al. Clinical safety of iron-fortified formulas. Pediatrics 2000;105(3):E38. doi: 10.1542/peds.105.3.e38 [published Online First: 2000/03/04]
12. Kennedy K, Fewtrell MS, Morley R, et al. Double-blind, randomized trial of a synthetic triacylglycerol in formula-fed term infants: effects on stool biochemistry, stool characteristics, and bone mineralization. The American journal of clinical nutrition 1999;70(5):920-7. doi: 10.1093/ajcn/70.5.920 [published Online First: 1999/10/28]
13. Fewtrell MS, Kennedy K, Murgatroyd PR, et al. Breast-feeding and formula feeding in healthy term infants and bone health at age 10 years. Br J Nutr 2013;110(6):1061-7. doi: 10.1017/s0007114512006149 [published Online First: 2013/02/08]
14. Fellegi IP, Sunter AB. A Theory for Record Linkage. Journal of the American Statistical Association 1969;64(328):1183-210. doi: 10.1080/01621459.1969.10501049
