# Supplementary material for: Infant formula composition and educational performance: a protocol to extend follow-up for a set of randomised controlled trials using linked administrative education records
Source: BMJ Open. 2020 Jul 23;10(7):e035968. doi: 10.1136/bmjopen-2019-035968 (PMC7380883; doi:10.1136/bmjopen-2019-035968)
Supplement: Author's manuscript [file bmjopen-2019-035968.draft_revisions.pdf]

BMJ Open is committed to open peer review. As part of this commitment we make the peer review history of every article we publish publicly available.

When an article is published we post the peer reviewers' comments and the authors' responses online. We also post the versions of the paper that were used during peer review. These are the versions that the peer review comments apply to.

The versions of the paper that follow are the versions that were submitted during the peer review process. They are not the versions of record or the final published versions. They should not be cited or distributed as the published version of this manuscript.

BMJ Open is an open access journal and the full, final, typeset and author-corrected version of record of the manuscript is available on our site with no access controls, subscription charges or pay-per-view fees (http://bmjopen.bmj.com).

If you have any questions on BMJ Open's open peer review process please email info.bmjopen@bmj.com

BMJ Open

Infant formula composition and educational performance: a protocol to extend follow-up for a set of randomised controlled trials using linked administrative education records

Journal:	*BMJ Open*
Manuscript ID	bmjopen-2019-035968
Article Type:	Protocol
Date Submitted by the Author:	25-Nov-2019
Complete List of Authors:	Verfürden, Maximiliane ; University College London, Great Ormond Street Institute of Child Health<br>Harron, Katie; University College London Institute of Child Health,<br>Jerrim, John; University College London, Institute of Education<br>Fewtrell, Mary; Great Ormond Street Hospital for Children,<br>Gilbert, Ruth; UCL Institute of Child Health, Centre for Paediatric Epidemiology and Biostatistics
Keywords:	Health policy < HEALTH SERVICES ADMINISTRATION & MANAGEMENT, Paediatric neurology < PAEDIATRICS, PUBLIC HEALTH, Nutrition < TROPICAL MEDICINE, Clinical trials < THERAPEUTICS

SCHOLARONE™
Manuscripts

I, the Submitting Author has the right to grant and does grant on behalf of all authors of the Work (as defined in the below author licence), an exclusive licence and/or a non-exclusive licence for contributions from authors who are: i) UK Crown employees; ii) where BMJ has agreed a CC-BY licence shall apply, and/or iii) in accordance with the terms applicable for US Federal Government officers or employees acting as part of their official duties; on a worldwide, perpetual, irrevocable, royalty-free basis to BMJ Publishing Group Ltd ("BMJ") its licensees and where the relevant Journal is co-owned by BMJ to the co-owners of the Journal, to publish the Work in this journal and any other BMJ products and to exploit all rights, as set out in our licence.

The Submitting Author accepts and understands that any supply made under these terms is made by BMJ to the Submitting Author unless you are acting as an employee on behalf of your employer or a postgraduate student of an affiliated institution which is paying any applicable article publishing charge ("APC") for Open Access articles. Where the Submitting Author wishes to make the Work available on an Open Access basis (and intends to pay the relevant APC), the terms of reuse of such Open Access shall be governed by a Creative Commons licence – details of these licences and which Creative Commons licence will apply to this Work are set out in our licence referred to above.

Other than as permitted in any relevant BMJ Author's Self Archiving Policies, I confirm this Work has not been accepted for publication elsewhere, is not being considered for publication elsewhere and does not duplicate material already published. I confirm all authors consent to publication of this Work and authorise the granting of this licence.

For peer review only

Protocol

Infant formula composition and educational performance: a protocol to extend follow-up for a set of randomised controlled trials using linked administrative education records

For peer review only

Authors:

Maximiliane L. Verfürden, *ESRC PhD student*[1] (m.verfuerden@ucl.ac.uk) *

Katie Harron, *Non-Clinical Senior Lecturer*[1,3] (k.harron@ucl.ac.uk)

John Jerrim, *Professor of Education and Social Statistics*[2] (j.jerrim@ucl.ac.uk)

Mary Fewtrell, *Professor of Paediatric Nutrition*[1] (m.fewtrell@ucl.ac.uk)

Ruth E Gilbert, *Professor of Clinical Epidemiology*[1,3] (r.gilbert@ucl.ac.uk)

[1]University College London Great Ormond Street Institute of Child Health

[2]University College London Institute of Education

[3]Health Data Research UK, London, United Kingdom

Correspondence to:

Maximiliane L. Verfuerden

Population, Policy and Practice Research & Teaching Department

UCL Great Ormond Street Institute of Child Health

30 Guilford Street London WC1N 1EH

Tel: 020 7905 2606

Manuscript word count: 2,304

Keywords: Infant Formula; Iron; Enrichment; Palmitate; Nucleotides; Docosahexaenoic acid; LCPUFA; Educational attainment; Cognition; Methodology; Post-trial; Randomized controlled trial; Long term; Follow-up

Acknowledgements: The authors thank the participants of the trials and their parents as well as the original trialists for the valuable time they have invested in generating this data.

For peer review only - http://bmjopen.bmj.com/site/about/guidelines.xhtml

Protocol approval: version 1.0

Authorised by:	*Date, Signature:*
Maximiliane L. Verfürden	*23 Nov 2019,*
Katie Harron	19 November 2019
John Jerrim	23 November 2019
Mary Fewtrell	*19th Nov 2019*
Ruth E. Gilbert	*27 Oct 2019,*

For peer review only

For peer review only - http://bmjopen.bmj.com/site/about/guidelines.xhtml

Abstract

Introduction

The effect of infant nutrition on long-term cognition is important for parents and policy makers. However, most clinical trials typically have short follow-up periods, when measures of cognition are poorly predictive of later function. The few trials with longer-term follow-up have very high levels of attrition, which can lead to selection bias, and in turn to erroneous interpretation of long-term harms and benefits of infant nutrition. We address the need for unbiased, long-term follow-up, by linking measures of educational performance from administrative education records. Educational performance is a meaningful marker of cognitive function in children and it is strongly correlated with intelligence quotient.We aim to evaluate educational performance for children who, as infants, were part of a series of trials that randomised participants to either nutritionally modified infant formula or standard formula. Most interventions anticipated positive effects on later cognitive function.

Methods and analysis

We will use record linkage between trial data and the National Pupil Database (NPD) to extend follow-up for a set of seven English infant formula trials, originally conducted between 1993 and 2001. Our primary outcome will be the mean differences in z-scores between intervention and control groups for a compulsory Mathematics exam sat at age 16. Secondary outcomes will be z-scores for a compulsory English exam at age 16 and z-scores for compulsory Mathematics and English exams at age 11. We will also evaluate intervention effects on the likelihood of receiving Special Educational Needs (SEN) support. All analyses will be performed separately by trial.

Ethics and dissemination

Research ethics approval, and approval from the Health Research Authority Confidentiality Advisory Group, has been obtained for this study. The results of this study will be disseminated to scientific, practitioner and lay audiences, submitted for publication in peer-reviewed journals, and will contribute towards a PhD dissertation.

For peer review only - http://bmjopen.bmj.com/site/about/guidelines.xhtml

Article Summary

Strengths and limitations of this study

- This study uses seven infant formula trials conducted in England to determine whether a range of nutritional interventions during infancy affects educational performance.
- We show that retrospective record linkage of trial to administrative data offers a low cost way to extend follow-up of early nutrition trials, maintain high rates of follow-up, and safeguard confidentiality.
- There is no agreement on what defines educational performance and at what age it is best measured to determine intervention effects. We therefore rely on data from two objectively assessed, compulsory examinations sat by the majority of state school students in England.

For peer review only

For peer review only - http://bmjopen.bmj.com/site/about/guidelines.xhtml

Introduction

Rationale

There is a need for high quality evidence on the long-term effects of infant formula on cognitive function to inform infant feeding recommendations and guide parents. However, the majority of randomised controlled trials (RCTs) report only short-term effects of formula composition on proxy end-points for cognition such as the Bayley Scales of Infant Development before two years of age.[1] Such early measures are crude, highly observer dependent and have poor predictive properties for later academic and employment outcomes. Better assessments of cognitive ability such as IQ scores are available during school age but are affected by high rates of loss to follow-up, which can bias results and reduce statistical power.[2]

Advances in the availability and quality of administrative datasets have created new possibilities for obtaining long-term outcomes on children's educational performance, which is a good predictor of future academic and employment opportunities.[3] Retrospective linkage of extant trial data to administrative educational performance data is achievable where trial data and participant identifiers have been retained and governance arrangements allow secure linkage without the need for participants to re-consent. Educational performance is highly correlated with IQ scores but socially more relevant than IQ scores as it determines future academic and employment opportunities. It is available for a fraction of the cost compared to setting up new RCTs or using traditional follow-up methods[4] and could offer a more rapid, complete, and therefore less biased way to determine long-term intervention effects.

We sought to determine long-term effects of modified infant formulas by using linkage of RCT records to educational performance data for seven existing trial cohorts that compared modified infant formula interventions with standard infant formula.

Primary objectives

- To determine the effect of nutritionally modified infant formula on educational performance in standardised Mathematics and English language tests at age 11 and 16 years.

For peer review only - http://bmjopen.bmj.com/site/about/guidelines.xhtml

Secondary objectives

- To assess whether infant formula modifications have an effect on the risk of receiving special educational needs support during school.
- To contribute data that can be used for future meta-analyses by exploring whether the effect of infant formula modifications on educational performance depends on maternal smoking status during pregnancy, birthweight or maturity at birth.

Methods

Data sources and linkage

We will follow participants from seven separate infant formula trials that randomised infants between 1993 and 2001 and were conducted in England (**Figure 1**). Full details on the individual trial cohorts as well as previously reported outcomes can be found elsewhere (**Supplementary Table 1**).

Outcome data for this follow-up will be retrieved from the National Pupil Database (NPD), an administrative data resource containing pupil-level and school-level data on all pupils in state schools in England and held by the Department for Education (DfE).[5] The Fischer Family Trust[6] identified pupil records for the trial participants in 2018 on the basis of identifying information collected at randomisation and during periods of active follow-up (**Figure 2**).

Interventions

The trials investigated the effect of seven different infant formula modifications in different infant populations (**Figure 3**). All but one of the interventions (sn-2 Palmitate) could plausibly have an effect on long-term cognitive function. The sn-2 Palmitate intervention formula cohort was included as a control cohort with an expected null-effect on educational performance. All analyses will be stratified by trial as effect mechanisms might differ.

Outcomes

Primary outcome

At age 16 years (during Key Stage 4), English pupils take their General Certificate of Secondary Education (GCSE). The primary outcome for this study will be the mean

For peer review only - http://bmjopen.bmj.com/site/about/guidelines.xhtml

difference in GCSE mathematics exam z-score between control and intervention groups. GCSE mathematics exams are compulsory, nationally administered and are graded from A*=58 points to G=16 or ungraded U=0 points.

We chose Mathematics over English, which is also compulsory and nationally administered, because exam results for mathematics are commonly considered to be less subjectively graded.[7] We chose the primary endpoint at age 16 rather than age 11 as it is a more relevant predictor of future education and employment opportunities. Children without KS4 will not be included in the primary outcome.

Secondary outcomes

As secondary outcomes we will investigate intervention effects of modified infant formula vs control formula on:

- mean GCSE English language exam z-scores
- mean Mathematics and English reading exam z-scores at age 11 (Key Stage 2, final year of primary school).
- probability of receiving five or more GCSEs with grades A* to C
- probability of receiving special educational needs (SEN) support

All mentioned Mathematics and English exams are compulsory and nationally administered. GCSE English language scores are graded like GCSE mathematic scores. Mathematics exams at age 11 are graded from 0–100, with 100 being highest possible score. English reading exams at age 11 are graded from 0–50, with 50 being highest. Receiving five or more GCSEs with grades A* to C is a commonly reported measure of academic performance as this measure feeds into entry requirements for a large number of sixth form colleges and therefore determines future academic development. Because the effect of the intervention on exam results might be mediated by special needs status, we also aim to determine whether the intervention affects the probability of receiving SEN support.

All z-scores will be calculated as:

$$\text{z-score} = \frac{x - \mu}{SD_{pooled}}$$

For peer review only - http://bmjopen.bmj.com/site/about/guidelines.xhtml

Where SD_{pooled} is the pooled standard deviation of the respective exam point scores from control and intervention groups within each trial.

Probabilities will be reported as odds of having the outcome in the intervention group over odds of having the outcome in the control group within each trial.

Covariates and interactions

To increase the statistical efficiency of our analysis we will adjust for covariates that have a strong association with the analysis outcome and were measured at randomisation (i.e. are not on the causal pathway between intervention and outcome). On the basis of previous internal analyses using linked *Millennium Cohort Study* and NPD data as well as previously published studies[3 8 9] we expect maternal smoking and maternal education as well as birthweight and gestational age to be strongly associated with educational performance. Analyses involving multi-centre trials with separate randomisation schedules for each centre will also be adjusted for centre. [10] However, not every covariate was measured in all trials (**Table 1**).

Table 1 Variables a-priori expected to be associated with educational performance

Trial<br>*Type*	Centre<br>*Nominal*	Gestational age<br>*Continuous*	Birthweight<br>*Continuous*	Maternal smoking<br>*Binary*	Maternal education<br>*Ordinal*
1993/94 Iron	X	X	X	X	X
1995/96 Sn-2 palmitate	n/a	X	X	–	–
2000/01 Nucleotides	n/a	X	X	–	X
1993/96 Nutrient-enriched post-dc.	X	X	X	X	X
1993/96 Nutrient-enriched (SGA)	X	X	X	X	X
1993/95 LCPUFA (term)	X	X	X	X	X
1993/96 LCPUFA (preterm)	X	X	X	X	X

n/a not applicable; '–' no data available; *LCPUFA* Long chain polyunsaturated fatty acids, *SGA* born small for gestational age, *post-dc.* formula after discharge from hospital

Based on reports from previous studies[11] we also hypothesise that the effect of supplementations will depend on sex and whether the mother smoked during pregnancy or not. However, the trials were not powered to measure this interaction; therefore, we will include an interaction term in an exploratory analysis so the results can be included in future meta-analyses.

For peer review only - http://bmjopen.bmj.com/site/about/guidelines.xhtml

Statistical methods

Primary analysis

For our primary analysis we will use multivariable regression models to compare intervention and control groups stratified by trial and adjusted for a-priori determined risk factors (**Table 1**). We will present effect estimates as mean differences with 95% confidence intervals. The primary analysis can be expressed using the following equation:

$$\text{Z-}score_{GCSE\ Maths} = \beta_0 + \beta_1 \times centre + \beta_2 \times sex + \beta_3 \times birthweight + \ldots + \beta_p \times control\ milk + e$$

Where β_0 (intercept) is the z-score of GCSE Mathematics when all predictors are zero and e refers to the residual terms. This model assumes that GCSE Mathematics scores are linearly related to the other predictors. If for a given predictor the relationship it is not linear, we will account for this by categorising quantitative variables or including quadratic terms. We will also check that there is no multicollinearity between the predictors, residuals are normally distributed and independent and that the variance is constant across the outcome and the predictors.

Secondary analyses

Mean GCSE English language exam z-scores, mean Mathematics and English reading exam z-scores at age 11 will be analysed in the same way as the primary outcome.
The probability of receiving five or more GCSEs with grades A* to C and the probability of receiving SEN support will be modelled use logistic regression, and adjusted for the same factors as the primary outcome (**Table 1**).

Sensitivity analyses

To determine the effect of the intervention on GCSE Mathematics scores in context of the general population, we will calculate the z-scores using national standard deviations rather than the trial specific ones. Due to the higher heterogeneity in the national sample, we expect our effect estimate to be more conservative in this analysis.

In order to assess the robustness of our analysis, we will also present the unadjusted outcomes as a sensitivity analysis, and model the outcome using an ordered logit model adjusting for the above mentioned covariates (**Table 1**).

For peer review only - http://bmjopen.bmj.com/site/about/guidelines.xhtml

To assess the sensitivity of our end-point measure, we are going to present results for the cohort of non-randomised breast-fed recruits from the trials alongside the randomised groups. Breastfed children have been consistently reported to have better cognitive ability and perform better in school than their formula-fed peers.[12 13] Therefore, we also expect to observe better school performance for previously breast-fed compared to formula-fed children in the primary outcome. Breastfed children were recruited in all trials except for the 1993/94 Iron trial. Additionally, we expect there to be no effect of sn-2 palmitate enriched formula and included this trial cohort to further validate our approach.

Exploratory analyses

While this study is underpowered to determine interaction effects, we intend to conduct exploratory analyses stratified by sex and smoking status during pregnancy so these are available for inclusion in future meta-analyses.

Power

The primary outcomes (mean difference in z-score of mathematics exam at age 16) will be continuous and analyses will be conducted for each trial separately. We expect primary outcomes to be available for around 70% of the original cohort with an equal retention rate in treatment and control arms. A 0.1 standard deviation (SD) difference[*] will be regarded as clinically significant.[14] The desired power to detect a difference between the treatment arms that is at least as large as this, is 80% with a significance level of 0.05. The R-squared is expected to be 0.05-0.1. Taking the 1993/95 LCPUFA term study (n=309) as an example, a conservative 50% (n=154) linkage success would enable a detection of 0.45 SD difference (e.g. 9/10 of a grade or 5.193 grade points).

Missing data

For our primary analysis we will use multiple imputation to impute missing covariates using 15 imputations and setting the random seed at 300.[10] We will use stata's *mi chained* command to perform the imputation. We will also perform sensitivity analyses to determine the potential impact of missing outcome data by looking at worst case scenarios (i.e. all

[*] this corresponds to 1/5th of a GCSE grade or 1.154 points on the 58 points point scale.

For peer review only - http://bmjopen.bmj.com/site/about/guidelines.xhtml

missing participants with missing outcome data achieved the lowest grade/ highest grade possible).

Assessment of bias

For each trial, we will compile information for a Cochrane risk of bias analysis.[15]
To detect whether selection bias at follow-up has affected the randomisation balance of characteristics we will present the pre-randomisation characteristics as percentage/ mean differences between intervention and control groups at randomisation and at age 16 (when the primary outcome is measured) with 95% confidence intervals.

Discussion

Expected outcomes of the study

High participant retention rates are essential to determine delayed intervention effects that might not become apparent in short-term follow-ups or when using traditional follow-up methods which typically suffer from high attrition. Extending follow-up of RCTs without re-consent is possible where trial data and participant identifiers have been retained and governance arrangements allow secure linkage that safeguards participants' privacy. Post-trial extension using unconsented linkage to administrative data will be especially desirable for future research if it reduces costs associated with conventional methods of follow-up (such as face-to face interviews) and simultaneously improves participant retention rates (compared to consented data linkage). Finally, this method will allow new insights into the long-term cognitive benefits and safety profile of modified infant formulas, by making use of unique historical trials which were comparing modified formulas to unsupplemented control formulas, which, are unlikely to be investigated in future RCTs either due to lack of equipoise or expense.

Limitations

Only schools following the national curriculum have to contribute data to the NPD, while independent schools may provide data voluntarily. Most participants who attended independent schools will therefore be excluded from this analysis.

Another limitation is that there is no general agreement on what defines educational performance and which age is optimal to capture intervention effects. To minimise missing

For peer review only - http://bmjopen.bmj.com/site/about/guidelines.xhtml

data and detect potential trajectory effects, we will therefore rely on data from two high-stakes examinations sat by the majority of state school students in England.

Finally, linkage certainty plays a role when using administrative data to follow up participants. The FFT provided us with up to three different pupil records per participants (based on names, date of birth and location). We used probabilistic methods adapted from Fellegi and Sunter[16] as well as manual review to decide which record, if any, we considered to be the best match (**Supplementary Material**).

Project Management

MV will clean and analyse the data and draft the initial manuscript. KH and RG will advise on data cleaning and contribute to the interpretation of results. JJ will contribute to interpreting exam results and MF will provide background information on the trials and contribute to interpreting potential effects of the modified formula. All authors will contribute to writing the final manuscript.

Dissemination of Results and Publication Policy

To enhance the use, understanding, and dissemination of this study, the results will be disseminated to both scientific and lay audiences, submitted for publication in peer-reviewed journals, and will contribute towards a PhD dissertation.

Funding

This work is supported by the Economic and Social Research Council (ESRC) and the Great Ormond Street Hospital Charity. RG and KH receive funding from Health Data Research UK, an initiative funded by the UK Research and Innovation, Department of Health and Social Care (England) and the devolved administrations, and leading medical research charities. KH receives funding from Wellcome (grant 103975/Z/14/Z). The funding sources had no role in study design, write-up or decision to publish. All authors had full access to all the data in the study and the corresponding author had final responsibility for the decision to submit for publication. All authors had full access to all the data in the study and the corresponding author had final responsibility for the decision to submit for publication.

For peer review only - http://bmjopen.bmj.com/site/about/guidelines.xhtml

Footnotes

Author Statement:

MV has prepared the initial draft of the manuscript. KH, JJ, MF and RG provided substantial intellectual input. All authors have contributed to writing the final draft of the manuscript.

Competing interests:

None declared.

Data sharing statement:

Statistical code will be available from GitHub. Data will be analysed under licence and are not available for sharing.

Ethics and patient consent:

This study has been approved by NHS Confidentiality Advisory Group (17CAG0051) and the London - City & East Research Ethics Committee (17/LO/0556). Patient consent for publication was not required.

Patient and public involvement:

We consulted with a subset of former trial participants about the acceptability of our study design and received strong support for our approach. We used the discussions to improve our reporting and dissemination strategy.

For peer review only

For peer review only - http://bmjopen.bmj.com/site/about/guidelines.xhtml

References

1. Sun H, Como PG, Downey LC, et al. Infant formula and neurocognitive outcomes: impact of study end-point selection. *Journal Of Perinatology* 2015;35:867. doi: 10.1038/jp.2015.87

https://www.nature.com/articles/jp201587#supplementary-information

2. Fewtrell MS, Domellof M, Hojsak I, et al. Attrition in Long-Term Nutrition Research Studies: A Commentary by the European Society for Pediatric Gastroenterology, Hepatology, and Nutrition Early Nutrition Research Working Group. *Journal of pediatric gastroenterology and nutrition* 2016;62(1):180-2. doi: 10.1097/mpg.0000000000000992 [published Online First: 2015/09/30]

3. Sammons P, Sylva K, Melhuish E, et al. Influences on students' GCSE attainment and progress at age 16. *Department for Education* 2014

4. Llewellyn-Bennett R, Edwards D, Roberts N, et al. Post-trial follow-up methodology in large randomised controlled trials: a systematic review. *Trials* 2018;19(1):298. doi: 10.1186/s13063-018-2653-0 [published Online First: 2018/05/31]

5. Jay MA, Mc Grath-Lone L, Gilbert R. Data Resource: the National Pupil Database (NPD). *International Journal of Population Data Science* 2019;4(1)

6. FFT Education Ltd. Fischer Family Trust 2019 [Available from: https://fft.org.uk/ accessed July 2019.

7. Rhead S, Black B. Marking consistency metrics: An update. In: Ofqual, ed. Coventry: Ofqual, 2018.

8. Botting N, Powls A, Cooke RW, et al. Cognitive and educational outcome of very-low-birthweight children in early adolescence. *Developmental Medicine & Child Neurology* 1998;40(10):652-60. doi: 10.1111/j.1469-8749.1998.tb12324.x

9. Abel K, Heuvelman H, Wicks S, et al. Gestational age at birth and academic performance: population-based cohort study. *International journal of epidemiology* 2017;46(1):324-35. doi: 10.1093/ije/dyw284 [published Online First: 2016/11/08]

10. European Medicines Agency. Guideline on Missing Data in Confirmatory Clinical Trials 2010.

11. de Jong C, Kikkert HK, Fidler V, et al. Effects of long-chain polyunsaturated fatty acid supplementation of infant formula on cognition and behaviour at 9 years of age. *Developmental medicine and child neurology* 2012;54(12):1102-8. doi: 10.1111/j.1469-8749.2012.04444.x [published Online First: 2012/10/17]

12. Victora CG, Horta BL, de Mola CL, et al. Association between breastfeeding and intelligence, educational attainment, and income at 30 years of age: a prospective birth cohort study from Brazil. *The Lancet Global Health* 2015;3(4):e199-e205. doi: 10.1016/S2214-109X(15)70002-1

For peer review only - http://bmjopen.bmj.com/site/about/guidelines.xhtml

13. Lucas A, Morley R, Cole TJ, et al. Breast milk and subsequent intelligence quotient in children born preterm. *Lancet (London, England)* 1992;339(8788):261-4. doi: 10.1016/0140-6736(92)91329-7 [published Online First: 1992/02/01]

14. Fryer Jr RG. The production of human capital in developed countries: Evidence from 196 randomized field experiments. Handbook of economic field experiments: Elsevier 2017:95-322.

15. Higgins JP, Altman DG, Gøtzsche PC, et al. The Cochrane Collaboration's tool for assessing risk of bias in randomised trials. *Bmj* 2011;343:d5928.

16. Fellegi IP, Sunter AB. A Theory for Record Linkage. *Journal of the American Statistical Association* 1969;64(328):1183-210. doi: 10.1080/01621459.1969.10501049

Figure legends/ captions

Figure 1: Combined flow of participants of seven infant formula trials (recruitment period: 1993-2001)

Figure 2: Linkage of trial data to administrative education data

Figure 3: Trial interventions, comparators and populations

For peer review only

For peer review only - http://bmjopen.bmj.com/site/about/guidelines.xhtml

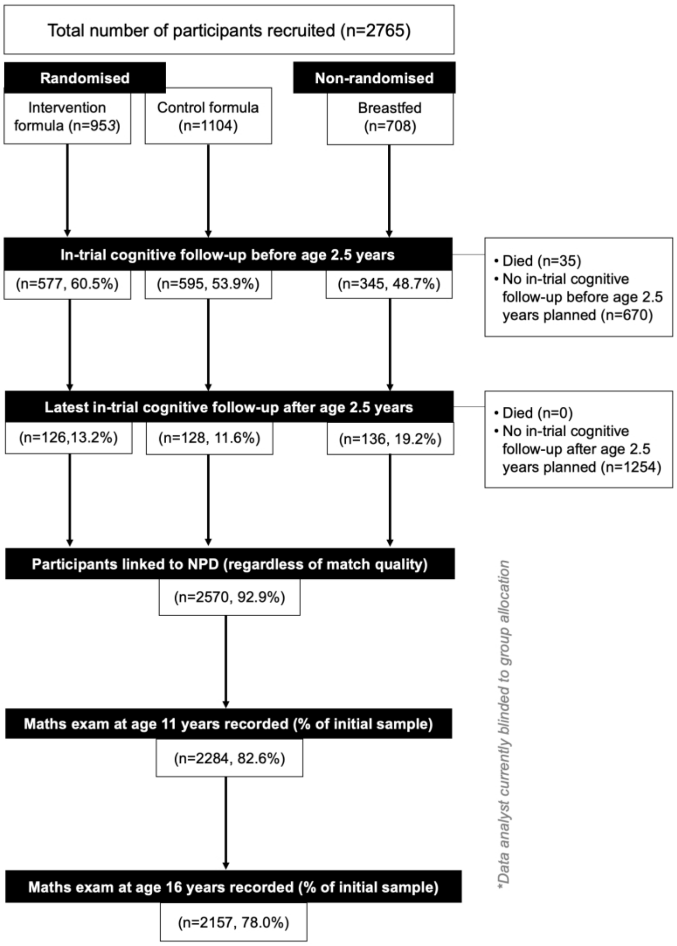

Figure 1 Participant flow

634x1057mm (72 x 72 DPI)

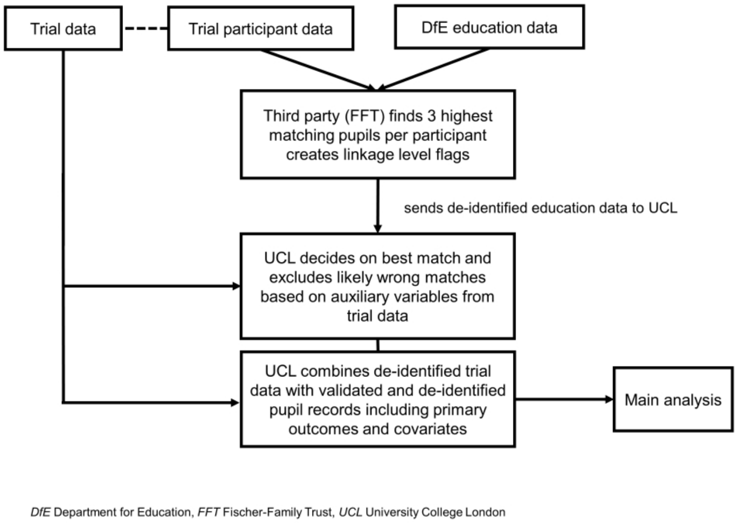

Figure 2 Data flow

351x230mm (300 x 300 DPI)

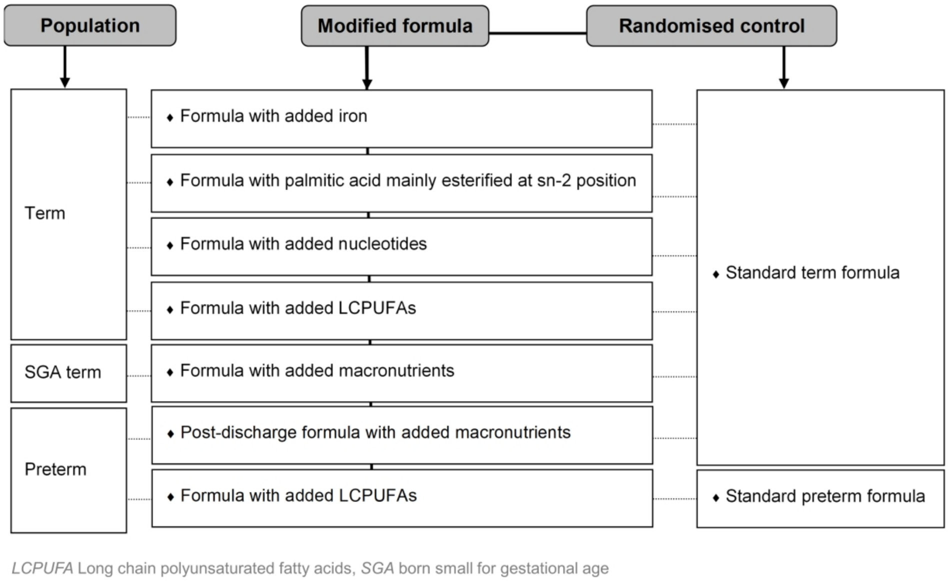

Figure 3 Trial interventions, comparators and population

351x230mm (300 x 300 DPI)

Supplementary material

Supplementary Table 1 Trial Characteristics

	Nutrient-enriched	Nutrient-enriched	LCPUFA	LCPUFA	Nucleotides	Iron	Sn-2 palmitate
**Population**	Preterm	SGA Term	Preterm	Term	Term	Term	Term
**Years randomised**	1993-96	1993-96	1993-96	1993-95	2000-01	1993-94	1995-96
**Eligibility**	<1750g bw<br><37 ga	bw <10th centile<br>≥37wga	≤1750g bw<br>≤37wga	≥37wga	≥37wga	bw > 2500g, ≥37wga<br>age 9m	≥37wga, bw>5th centile
**Control formula:**	Term formula	Term formula	Preterm formula	Term formula	Term formula	Term formula<br>or cow's milk	Term formula
**Intervention:**							
Control formula with added:	Macro nutrients and vitamins	Macro nutrients and vitamins	DHA and AA	DHA and AA	Nucleotides	12 mg/L iron	Sn-2 palmitate
**Timing of intervention**	1w < discharge - 9m	Birth – 12w	Birth – 3w	Birth – 6m	Birth – 20w	9m - 18m	Birth – 12w
**Centres**	Nottingham<br>Leicester<br>Cambridge<br>Ipswich	Nottingham<br>Leicester<br>Cambridge	Nottingham<br>Leicester	Nottingham<br>Leicester	Nottingham<br>Leicester	Nottingham<br>Leicester<br>Norwich	Cambridge
**Total nr. randomised**	245	325	219	335	205	512	217
Intervention	123	168	110	166	103	171	113
Control	122	157	109	169	102	341	104
**Nr. non-randomised breastfed reference**	69	182	89	142	105	0	121
**Reference nr.**	[17]	[18,19]	[20,21]	[22,23]	[24,25]	[26,27]	[28,29]

bw=birthweight, ga=gestational age, w=weeks, LCPUFA=Long Chain Polyunsaturated Fatty Acids, sga=small-for-gestational-age, m=months, d=days, Notth=Nottingham, Cambr=Cambridge, Ipsw= Ipswich, Leics=Leicester, cent=centile,

For peer review only

Data cleaning methods

The party who conducts the linkage (the Fischer Family Trust, FFT) provide up to three likely matching pupil IDs and their school records per participant. Only one of these records can be a true match and it is possible that none of the linked pupil IDs is a true match, as participants might have emigrated, died or attended private schools which do not transmit data to the national pupil database. We will check and cross validate the data provided by FFT extensively, using auxiliary variables from the trials (such as known death) and linkage confidence flags supplied by FFT (**Supplementary Table 2**).

Supplementary Table 2 Possible match levels

Identifier	Description
**First name**	First name and other first name both exact match<br>First name matches other name in both directions<br>First name exact match<br>First name matches other name<br>Other name exact match<br>First name truncated at any hyphen matches<br>First name matches via common name alternatives lookup<br>Pattern match function<br>Pattern match function - AND first character of first name matches<br>First name / surname match in both directions<br>No Link
**Surname**	Surname exact match (including alternative surnames)<br>Surname truncated at any hyphen matches<br>Pattern match function<br>Pattern match function - AND first character of surname matches<br>First name / surname match in both directions<br>No Link
**Date of Birth**	DOB exact match<br>Day on source matches month on match, and vice versa; year matches (i.e. transposed date)<br>Day and month match (i.e. wrong year)<br>Day and year match (i.e. wrong month)<br>Month and year match (i.e. wrong day)<br>Either source or match DOB is 1st January; year matches<br>Either source or match DOB is 1st September; year matches<br>No Link
**Location**	Postcode exact match<br>Local Authority match<br>Neighbouring / nearby Local Authority match<br>No Link

We will calculate match-weights for each participant-pupil pair according to a probabilistic method adapted from Fellegi and Sunter.[16] These match-weights will then be used to rank the

For peer review only - http://bmjopen.bmj.com/site/about/guidelines.xhtml

pairs according to their probability of being a true match. For each match category (**Supplementary Table 2**) we will derive proxy-m and proxy-u probabilities which will then be used to produce the match weight.

M-probability: probability of agreeing on a category given that it is a true match
U-probability: probability of agreeing on a category given by chance

The original method requires a set of participant-pupil pairs that are certain true matches and a set of participant-pupil pairs that are certain false matches. Due to the de-identified nature of the data, such information will not be available and thus needs to be approximated. True matches will be defined as participants perfectly matching all categories (except the one we are calculating the probability for). Certain false matches will be defined as participants disagreeing in all categories (except the one we are calculating the probability for). This will yield a total of eight reference datasets. For each linkage flag (first name, last name, date of birth, location) we will then calculate the variable weight using:

$$\mathbf{log2\ (m/u)}$$

The final match weight for a participant-pupil pair will be calculated using the sum of all weights in a pair:

$$\sum \mathbf{log2\ (m/u)}$$

At this stage each participant will still have up to three multiple possible matches.
First, we will drop all participants that linked but where their age at first link was >21. This is because the date of birth in the trial data is very likely to be correct as it corresponds to the recruitment periods and was confirmed repeatedly during the follow-up period. We will also discard all records for children that died before school age (4 years) where information on death and time of death during the trial period is available.

We will then rank participant-pupil pairs according to their match weights. If the highest match-weight for a participant is 10% above that of the second best match, we will automatically keep the best match and discard the other(s). If the difference is lower, we will manually review these records to decide which one is the best match (if any). All decisions taken at manual review will be reported to ensure reproducibility. We will then choose two cut-offs based on visual inspection of the match probabilities; one threshold to maximise sensitivity (many true positive links) and one to maximise specificity (few false positive links). To determine how representative each sample defined by these cut-offs is of the

For peer review only

original randomised cohort, we will compare them across the variables that were measured at randomisation. We will use the bigger, more sensitive sample in our analyses but use the smaller, more specific sample in sensitivity analyses.

For peer review only

BMJ Open

Infant formula composition and educational performance: a protocol to extend follow-up for a set of randomised controlled trials using linked administrative education records

Journal:	*BMJ Open*
Manuscript ID	bmjopen-2019-035968.R1
Article Type:	Protocol
Date Submitted by the Author:	01-May-2020
Complete List of Authors:	Verfürden, Maximiliane ; University College London, Great Ormond Street Institute of Child Health<br>Harron, Katie; University College London Institute of Child Health,<br>Jerrim, John; University College London, Institute of Education<br>Fewtrell, Mary; Great Ormond Street Hospital for Children,<br>Gilbert, Ruth; UCL Institute of Child Health, Centre for Paediatric Epidemiology and Biostatistics
**Primary Subject Heading**:	Nutrition and metabolism
Secondary Subject Heading:	Health informatics, Paediatrics, Public health
Keywords:	Paediatric neurology < PAEDIATRICS, PUBLIC HEALTH, Clinical trials < THERAPEUTICS, NUTRITION & DIETETICS

SCHOLARONE™
Manuscripts

I, the Submitting Author has the right to grant and does grant on behalf of all authors of the Work (as defined in the below author licence), an exclusive licence and/or a non-exclusive licence for contributions from authors who are: i) UK Crown employees; ii) where BMJ has agreed a CC-BY licence shall apply, and/or iii) in accordance with the terms applicable for US Federal Government officers or employees acting as part of their official duties; on a worldwide, perpetual, irrevocable, royalty-free basis to BMJ Publishing Group Ltd ("BMJ") its licensees and where the relevant Journal is co-owned by BMJ to the co-owners of the Journal, to publish the Work in this journal and any other BMJ products and to exploit all rights, as set out in our licence.

The Submitting Author accepts and understands that any supply made under these terms is made by BMJ to the Submitting Author unless you are acting as an employee on behalf of your employer or a postgraduate student of an affiliated institution which is paying any applicable article publishing charge ("APC") for Open Access articles. Where the Submitting Author wishes to make the Work available on an Open Access basis (and intends to pay the relevant APC), the terms of reuse of such Open Access shall be governed by a Creative Commons licence – details of these licences and which Creative Commons licence will apply to this Work are set out in our licence referred to above.

Other than as permitted in any relevant BMJ Author's Self Archiving Policies, I confirm this Work has not been accepted for publication elsewhere, is not being considered for publication elsewhere and does not duplicate material already published. I confirm all authors consent to publication of this Work and authorise the granting of this licence.

For peer review only

Protocol bmjopen-2019-035968

Infant formula composition and educational performance: a protocol to extend follow-up for a set of randomised controlled trials using linked administrative education records

Authors:

Maximiliane L. Verfürden, *PhD student*[1] (m.verfuerden@ucl.ac.uk) *

Katie Harron, *Non-Clinical Senior Lecturer*[1,3] (k.harron@ucl.ac.uk)

John Jerrim, *Professor of Education and Social Statistics*[2] (j.jerrim@ucl.ac.uk)

Mary Fewtrell, *Professor of Paediatric Nutrition*[1] (m.fewtrell@ucl.ac.uk)

Ruth E Gilbert, *Professor of Clinical Epidemiology*[1,3] (r.gilbert@ucl.ac.uk)

[1]University College London Great Ormond Street Institute of Child Health

[2]University College London Institute of Education

[3]Health Data Research UK, London, United Kingdom

Correspondence to:

Maximiliane L. Verfuerden

Population, Policy and Practice Research & Teaching Department

UCL Great Ormond Street Institute of Child Health

30 Guilford Street London WC1N 1EH

Tel: 020 7905 2606

Manuscript word count: 3,001

Acknowledgements: The authors thank the participants of the trials and their parents as well as the original investigators for the valuable time they have invested in generating this data. We also thank the peer reviewers for their valuable comments, which in our view have significantly improved the quality of our protocol.

For peer review only - http://bmjopen.bmj.com/site/about/guidelines.xhtml

Abstract

Introduction

The effect of infant nutrition on long-term cognition is important for parents and policy makers. However, most clinical trials typically have short follow-up periods, when measures of cognition are poorly predictive of later function. The few trials with longer-term follow-up have high levels of attrition, which can lead to selection bias, and in turn to erroneous interpretation of long-term harms and benefits of infant nutrition. We address the need for unbiased, long-term follow-up, by linking measures of educational performance from administrative education records. Educational performance is a meaningful marker of cognitive function in children and it is strongly correlated with intelligence quotient. We aim to evaluate educational performance for children who, as infants, were part of a series of trials that randomised participants to either nutritionally modified infant formula or standard formula. Most interventions anticipated positive effects on later cognitive function.

Methods and analysis

Using data from 1,923 participants of seven randomised infant formula trials linked to the English National Pupil Database (NPD), this study will provide new insights into the effect of nutrient intake in infancy on school achievement. Our primary outcome will be the mean differences in z-scores between intervention and control groups for a compulsory Mathematics exam sat at age 16. Secondary outcomes will be z-scores for a compulsory English exam at age 16 and z-scores for compulsory Mathematics and English exams at age 11. We will also evaluate intervention effects on the likelihood of receiving Special Educational Needs (SEN) support. All analyses will be performed separately by trial.

Ethics and dissemination

Research ethics approval, and approval from the Health Research Authority Confidentiality Advisory Group, has been obtained for this study. The results of this study will be disseminated to scientific, practitioner and lay audiences, submitted for publication in peer-reviewed journals, and will contribute towards a PhD dissertation.

For peer review only - http://bmjopen.bmj.com/site/about/guidelines.xhtml

Article Summary

Strengths and limitations of this study

- This study uses seven infant formula trials conducted in England to determine whether a range of nutritional interventions during infancy affects educational performance.
- We show that retrospective record linkage of trial to administrative data offers a low cost way to extend follow-up of early nutrition trials, maintain high rates of follow-up, and safeguard confidentiality.
- There is no agreement on what defines educational performance and at what age it is best measured to determine intervention effects. We therefore rely on data from two objectively assessed, compulsory examinations sat by the majority of state school students in England.

For peer review only - http://bmjopen.bmj.com/site/about/guidelines.xhtml

Introduction

Rationale

There is a need for high quality evidence on the long-term effects of infant formula on cognitive function to inform infant feeding recommendations and guide parents. However, the majority of randomised controlled trials (RCTs) report only short-term effects of formula composition on proxy end-points for cognition such as the Bayley Scales of Infant Development before two years of age.[1] Such early measures are crude, highly observer dependent and have poor predictive properties for later academic and employment outcomes.[2] Better assessments of cognitive ability such as IQ scores are available during school age but are affected by high rates of loss to follow-up, which can bias results and reduce statistical power.[3]

Advances in the availability and quality of administrative datasets have created new possibilities for obtaining long-term outcomes on children's educational performance, which is a good predictor of future academic and employment opportunities.[4] Retrospective linkage of extant trial data to administrative educational performance data is achievable where trial data and participant identifiers have been retained and governance arrangements allow secure linkage without the need for participants to re-engage. Educational performance is highly correlated with IQ scores[5] but socially more relevant than IQ scores as it determines future academic and employment opportunities.[6] Linkage to administrative records is available for a fraction of the cost compared to setting up new RCTs or using traditional follow-up methods[7] and could offer a more rapid, complete, and therefore less biased way to determine long-term intervention effects.

We sought to determine long-term effects of modified infant formulas by linking RCTs to educational performance data for seven existing trial cohorts that compared modified infant formula interventions with standard infant formula.

Research objective 1

- To determine the effect of nutritionally modified infant formula on educational performance in standardised Mathematics and English language tests at age 11 and 16 years.

For peer review only

Research objective 2

- To assess whether infant formula modifications have an effect on the risk of receiving special educational needs support during school.

Research objective 3

- To explore whether the effect of infant formula modifications on educational performance differs for boys and girls, by maternal smoking status during pregnancy, birthweight, or maturity at birth.

Methods

Data sources and linkage

We will follow 1,923 participants from seven separate infant formula trials that randomised infants between 1993 and 2002 and were conducted in England (**Figure 1**). Full details on the individual trial cohorts as well as previously reported outcomes can be found elsewhere (**Supplementary Table 1**).

Outcome data for this follow-up will be retrieved from the National Pupil Database (NPD), an administrative data resource containing pupil-level and school-level data on all pupils in state schools in England and held by the Department for Education (DfE).[8] The Fischer Family Trust[9] (FFT) identified pupil records for the trial participants in 2018 on the basis of identifying information collected at randomisation and during periods of active follow-up (**Figure 2, Supplementary Table 2**).

Interventions

The seven parallel group trials investigated the superiority of different infant formula modifications over standard formula in different infant populations (**Figure 3**). The formula compositions for each trial are detailed in the supplementary material. All but two of the interventions (sn-2 Palmitate and Nucleotides) were assumed to have an effect on long-term cognitive function. All analyses will be stratified by trial as effect mechanisms might differ.

For peer review only - http://bmjopen.bmj.com/site/about/guidelines.xhtml

Outcomes

Primary outcome

At age 16 years (during Key Stage 4), English pupils take their General Certificate of Secondary Education (GCSE). The primary outcome for this study will be the mean difference in GCSE mathematics exam z-score between control and intervention groups. GCSE mathematics exams are compulsory, nationally administered and are graded from 58 points to 0 points (**Table 1**).

Table 1 GCSE Grade Structure during analysis period (2001-2016)

Grade	Points equivalent	Remarks
A*	58	Highest pass
A	32	
B	46	
C	40	
D	34	
E	28	
F	22	
G	16	Lowest pass
U	0	Ungraded

We chose Mathematics over English, which is also compulsory and nationally administered, because exam results for mathematics are commonly considered to be less subjectively graded.[10] We chose the primary endpoint at age 16 rather than age 11 as it is a more relevant predictor of future education and employment opportunities. Children without KS4 will not be included in the primary outcome.

Secondary outcomes

As secondary outcomes we will investigate intervention effects of modified infant formula vs control formula on:

- mean GCSE English language exam z-scores
- mean Mathematics and English reading exam z-scores at age 11 (Key Stage 2, final year of primary school).

For peer review only - http://bmjopen.bmj.com/site/about/guidelines.xhtml

- probability of receiving five or more GCSEs with grades A* to C which includes Mathematics and English. (This is a critical accountability measure for schools, and is used in school performance tables).
- probability of receiving special educational needs (SEN) support

All mentioned Mathematics and English exams are compulsory and nationally administered. GCSE English language scores are graded like GCSE mathematic scores. Mathematics exams at age 11 are graded from 0–100, with 100 being highest possible score. English reading exams at age 11 are graded from 0–50, with 50 being highest. Receiving five or more GCSEs with grades A* to C is a commonly reported measure of academic performance as this measure feeds into entry requirements for a large number of sixth form colleges and therefore determines future academic development. Because the effect of the intervention on exam results might be mediated by special needs status, we also aim to determine whether the intervention affects the probability of receiving SEN support.

All z-scores will be calculated as:

$$\text{z-score} = \frac{x - \mu}{SD_{pooled}}$$

Where SD_{pooled} is the pooled standard deviation of the respective exam point scores from control and intervention groups within each trial.

Probabilities will be reported as odds of having the outcome in the intervention group over odds of having the outcome in the control group within each trial.

Covariates and interactions

To increase the statistical efficiency of our analysis we will adjust for covariates that have a strong association with the analysis outcome and were measured at randomisation (i.e. are not on the causal pathway between intervention and outcome). On the basis of previous internal analyses using linked *Millennium Cohort Study* and NPD data as well as previously published studies[4 11 12] we expect maternal smoking and maternal education as well as birthweight and gestational age to be strongly associated with educational performance. Analyses involving multi-centre trials with separate randomisation schedules for each centre will also be adjusted for centre. [13] However, not every covariate was measured in all trials (**Table 2**).

For peer review only - http://bmjopen.bmj.com/site/about/guidelines.xhtml

Table 2 Variables a-priori expected to be associated with educational performance

Trial	Centre	Gestational age	Birthweight	Maternal smoking	Maternal education
Type	*Nominal*	*Continuous*	*Continuous*	*Binary*	*Ordinal*
RCT 1 1993/96 Nutrient-enriched post-dc.	X	X	X	X	X
RCT 2 1993/96 Nutrient-enriched (SGA)	X	X	X	X	X
RCT 3 1993/96 LCPUFA (preterm)	X	X	X	X	X
RCT 4 1993/95 LCPUFA (term)	X	X	X	X	X
RCT 5 2000/02 Nucleotides	X	X	X	X	X
RCT 6 1993/94 Iron	X	X	X	X	X
RCT 7 1995/96 Sn-2 palmitate	n/a	X	X	X	X

n/a not applicable; *LCPUFA* Long chain polyunsaturated fatty acids, *SGA* born small for gestational age, *post-dc.* formula after discharge from hospital

Based on reports from previous studies[14] we also hypothesise that the effect of supplementations will depend on sex and whether the mother smoked during pregnancy or not. However, the trials were not powered to measure this interaction; therefore, we will include an interaction term in an exploratory analysis so the results can be included in future meta-analyses.

Statistical methods

All statistical analyses will be conducted using Stata version 16.[15]

Primary analysis

For our primary analysis we will use multivariable regression models to compare intervention and control groups stratified by trial and adjusted for a-priori determined risk factors (**Table 2**). We will present effect estimates as mean differences with 95% confidence intervals. The analysis will be on intention-to-treat basis. The primary analysis can be expressed using the following equation:

For peer review only - http://bmjopen.bmj.com/site/about/guidelines.xhtml

$$\text{Z-}score_{GCSE\ Maths} = \beta_0 + \beta_1 \times centre + \beta_2 \times sex + \beta_3 \times birthweight + \ldots + \beta_p \times control\ milk + e$$

Where β_0 (intercept) is the z-score of GCSE Mathematics when all predictors are zero and e refers to the residual terms. This model assumes that GCSE Mathematics scores are linearly related to the other predictors. If for a given predictor the relationship it is not linear, we will account for this by categorising quantitative variables or including quadratic terms. We will also check that there is no multicollinearity between the predictors, residuals are normally distributed and independent and that the variance is constant across the outcome and the predictors.

Secondary analyses

Mean GCSE English language exam z-scores, and mean Mathematics and English reading exam z-scores at age 11 will be analysed in the same way as the primary outcome.
The probability of receiving five or more GCSEs with grades A* to C and the probability of receiving SEN support will be modelled use logistic regression, and adjusted for the same factors as the primary outcome (**Table 2**). We will take into account the risk of false positives arising from multiple testing when interpreting the results.

Sensitivity analyses

To determine the effect of the intervention on GCSE Mathematics scores in context of the general population, we will calculate the z-scores using national standard deviations rather than the trial specific ones. Due to the higher heterogeneity in the national sample, we expect our effect estimate to be more conservative in this analysis.
In order to assess the robustness of our analysis, we will also present the unadjusted outcomes as a sensitivity analysis, and model the outcome using an ordered logit model adjusting for the above mentioned covariates (**Table 2**).
To assess the sensitivity of our end-point measure, we are going to present results for the cohort of non-randomised breast-fed recruits from the trials alongside the randomised groups. Breastfed children have been consistently reported to have better cognitive ability and perform better in school than their formula-fed peers.[16,17] Therefore, we also expect to observe better school performance for previously breast-fed compared to formula-fed children in the primary outcome. Breastfed children were recruited in all trials except for the

For peer review only - http://bmjopen.bmj.com/site/about/guidelines.xhtml
For peer review only

1993/94 Iron trial. Additionally, we expect there to be no effect of sn-2 palmitate enriched formula and included this trial cohort to further validate our approach.

Exploratory analyses

While this study is underpowered to determine interaction effects, we intend to conduct exploratory analyses stratified by sex and smoking status during pregnancy, so these are available for inclusion in future meta-analyses.

Power

The primary outcomes (mean difference in z-score of mathematics exam at age 16) will be continuous and analyses will be conducted for each trial separately. We expect primary outcomes to be available for around 70% of the original cohort with an equal retention rate in treatment and control arms. A 0.1 standard deviation (SD) difference* will be regarded as clinically significant.[18 19] The desired power to detect a difference between the treatment arms that is at least as large as this, is 80% with a significance level of 0.05. The R-squared is expected to be 0.05-0.1. Taking the 1993/95 LCPUFA term study (n=309) as an example, a conservative 50% (n=154) linkage success would enable a detection of 0.45 SD difference (e.g. 9/10 of a grade or 5.193 grade points).

Missing data

For our primary analysis we will use multiple imputation to impute missing covariates using 15 imputations and setting the random seed at 300.[13] We will use Stata's *mi chained* command to perform the imputation. We will also perform sensitivity analyses to determine the potential impact of missing outcome data by looking at worst case scenarios (i.e. all missing participants with missing outcome data achieved the lowest grade/ highest grade possible).

Assessment of bias

For each trial, we will compile information for a Cochrane risk of bias analysis.[20]

To detect whether selection bias at follow-up has affected the randomisation balance of characteristics we will present the pre-randomisation characteristics as percentage/ mean

* this corresponds to 1/5th of a GCSE grade or 1.154 points on the 58 points point scale.

For peer review only - http://bmjopen.bmj.com/site/about/guidelines.xhtml

differences between intervention and control groups at randomisation and at age 16 (when the primary outcome is measured) with 95% confidence intervals.

Discussion

Expected outcomes of the study

High participant retention rates are essential to determine delayed intervention effects that might not become apparent in short-term follow-ups or when using traditional follow-up methods which typically suffer from high attrition.[3 21] Extending follow-up of RCTs without the need for participants to re-engage is possible where trial data and participant identifiers have been retained and governance arrangements allow secure linkage that safeguards participants' privacy. Post-trial extension using opt-out linkage to administrative data will be especially desirable for future research if it reduces costs associated with conventional methods of follow-up (such as face-to face interviews) and simultaneously improves participant retention rates (compared to opt-in data linkage). Critically, our study will allow new insights into the long-term cognitive benefits and safety profile of modified infant formulas, by making use of randomised historical infant formula comparisons which, are unlikely to be investigated in future RCTs either due to lack of equipoise or resources.

Limitations

Our study should be considered in the context of several limitations.

First, only schools following the national curriculum have to contribute data to the NPD, while independent schools may provide data voluntarily. [22] Some participants who received their education outside state-funded English schools will therefore be excluded from this analysis. In any given academic year, however, data from 99% of all children of compulsory school age are included in the NPD database [8]. Consequently, we expect this to have minimal, non-differential impact on overall linkage success.

Second, there is no general agreement on what defines educational performance and which age is optimal to capture intervention effects. To minimise missing data and detect potential trajectory effects, we will therefore rely on data from two high-stakes examinations sat by the majority of state school students in England.

For peer review only - http://bmjopen.bmj.com/site/about/guidelines.xhtml

Third, linkage quality plays a role when using administrative data to follow up participants.[23-26] The FFT will provide us with up to three different pupil records per participants (based on names, date of birth and location). We will use probabilistic methods adapted from Fellegi and Sunter[27] as well as manual review to decide which record, if any, we consider to be the best match (**Supplementary Material**).

Fourth, we acknowledge that intervention effects measured 15 years later might be diluted by contextual factors. Convergence of trial arms may result from catch-up over time as those who lag behind in early years might receive extra (e.g. educational) assistance. There will also be variability in terms of quality of education. However, this is likely to be roughly equal between intervention and control groups due to adequate sample size, randomisation, and blinding. Furthermore, theories of early programming suggest that early postnatal life is a critical period of development[28] and nutritional stimuli during this period can result in lifelong permanent effects[29]. If true, any effective nutritional intervention is unlikely to disappear completely by early adulthood. If not, our study should be of increased interest to the scientific community concerned with the early origins of adult health and disease theory. Either way, it will provide important insights for parents, policy makers, and the scientific community as to whether choice of infant formula type can have long-lasting, measurable effects on something as important as educational performance.

Ethics and dissemination

Ethics approval and legal basis for data processing

The proposal to link the trials and NHS data underwent a robust ethical, legal, and technical review. The proposed project was approved by the UCL Research & Development Office and Data Protection Office (Reference: 14PE15), the NHS London-City & East Research Ethics Committee (Reference: 17/LO/0556) as well as the Health Research Authority and Health and Care Research Wales (Reference: 212148). An exemption under Section 251 of the NHS Act 2006 was granted by the Confidentiality Advisory Group (Reference: 17CAG0051) and allows UCL GOS ICH and the FFT to process identifiable participant information to facilitate the data linkage and analyses without consent. Dissent and fair processing were discussed with the HRA CAG and an opt-out procedure is in place.[30]

Confidentiality

For peer review only - http://bmjopen.bmj.com/site/about/guidelines.xhtml

Special efforts are made protect participant privacy and confidentiality. All linked data will be de-identified and thus contain no information on personal identifiers such as names and addresses. Identifiers will be stored at separate access restricted locations certified to international standards (ISO/IEC 27001). Access to all data will also be physically restricted. Analysis data will be stored as aggregated as possible (such as school types instead of school IDs), to minimise the risk of re-identification. All analyses will be conducted in a digital safe haven and no individual level data is allowed to leave the server. Outputs will have to meet statistical disclosure controls that prevent small cell sizes to be exported from the server. Additionally, access to the data will be restricted to an authorised group of researchers, who will all have undergone the HSCIC and ADRN information governance training and are bound by their university contracts to treat the data confidentially.

Project management

The Fischer Family Trust links the data which is then securely transferred to the UCL data safe haven. MV will clean and analyse the linked data and draft the initial manuscript. KH and RG will advise on data cleaning and contribute to the interpretation of results. JJ will contribute to interpreting exam results and MF will provide background information on the trials and contribute to interpreting potential effects of the modified formula. All authors will contribute to writing the final manuscript.

Dissemination of results and publication policy

To enhance the use, understanding, and dissemination of this study, the results will be disseminated to both scientific and lay audiences, submitted for publication in peer-reviewed journals, and will contribute towards a PhD dissertation.

Funding

MV is supported by the Economic and Social Research Council (ESRC) and the Great Ormond Street Hospital Charity. RG and KH receive funding from Health Data Research UK, an initiative funded by the UK Research and Innovation, Department of Health and Social Care (England) and the devolved administrations, and leading medical research charities. KH receives funding from Wellcome (grant 103975/Z/14/Z). The funding sources had no role in study design, write-up or decision to publish.

For peer review only - http://bmjopen.bmj.com/site/about/guidelines.xhtml

Footnotes

Author Statement:

MV has prepared the initial draft of the protocol. KH, JJ, MF and RG provided substantial intellectual input. All authors have contributed to writing the final draft of the manuscript.

Competing interests:

Mary Fewtrell has been a member of the Infant Nutrition Working Group at EFSA (European Food Safety Authority) since 2013. She was involved in data analysis and publication of randomised trials of LCPUFA-supplemented infant formulas funded by grants from Numico Res BV and Heinz UK. The companies also provided the infant formulas for the studies. She was also involved in follow-up studies (including cognitive outcome) of children and adolescents from randomised trials of LCPUFA-supplemented formulas, with funding from the Medical Research Council and European Union (FP6-FOOD-2005-007036). Maximiliane Verfuerden, Katie Harron, John Jerrim, and Ruth Gilbert have no competing interests to declare.

Data sharing statement:

Statistical code will be available from GitHub. Data will be analysed under licence and are not available for sharing. Aggregate, non-identifiable data are available upon reasonable request.

Ethics and patient consent:

This study has been approved by NHS Confidentiality Advisory Group (17CAG0051) and the London - City & East Research Ethics Committee (17/LO/0556). Patient consent for publication was not required.

Patient and public involvement:

We consulted with a subset of former trial participants about the acceptability of our study design and received strong support for our approach. We used the discussions to improve our reporting and dissemination strategy.

For peer review only - http://bmjopen.bmj.com/site/about/guidelines.xhtml

References

1. Sun H, Como PG, Downey LC, et al. Infant formula and neurocognitive outcomes: impact of study end-point selection. *Journal Of Perinatology* 2015;35:867. doi: 10.1038/jp.2015.87
https://www.nature.com/articles/jp201587#supplementary-information

2. Sun H, Como PG, Downey LC, et al. Infant formula and neurocognitive outcomes: Impact of study end-point selection. *Journal of Perinatology* 2015;35(10):867-74.

3. Fewtrell MS, Domellof M, Hojsak I, et al. Attrition in Long-Term Nutrition Research Studies: A Commentary by the European Society for Pediatric Gastroenterology, Hepatology, and Nutrition Early Nutrition Research Working Group. *Journal of pediatric gastroenterology and nutrition* 2016;62(1):180-2. doi: 10.1097/mpg.0000000000000992 [published Online First: 2015/09/30]

4. Sammons P, Sylva K, Melhuish E, et al. Influences on students' GCSE attainment and progress at age 16. *Department for Education* 2014

5. Deary IJ, Strand S, Smith P, et al. Intelligence and educational achievement. *Intelligence* 2007;35(1):13-21.

6. Borghans L, Golsteyn BHH, Heckman JJ, et al. What grades and achievement tests measure. *Proceedings of the National Academy of Sciences of the United States of America* 2016;113(47):13354-59. doi: 10.1073/pnas.1601135113 [published Online First: 2016/11/08]

7. Llewellyn-Bennett R, Edwards D, Roberts N, et al. Post-trial follow-up methodology in large randomised controlled trials: a systematic review. *Trials* 2018;19(1):298. doi: 10.1186/s13063-018-2653-0 [published Online First: 2018/05/31]

8. Jay MA, Mc Grath-Lone L, Gilbert R. Data Resource: the National Pupil Database (NPD). *International Journal of Population Data Science* 2019;4(1)

9. FFT Education Ltd. Fischer Family Trust 2019 [Available from: https://fft.org.uk/ accessed July 2019.

10. Rhead S, Black B. Marking consistency metrics: An update. In: Ofqual, ed. Coventry: Ofqual, 2018.

11. Botting N, Powls A, Cooke RW, et al. Cognitive and educational outcome of very-low-birthweight children in early adolescence. *Developmental Medicine & Child Neurology* 1998;40(10):652-60. doi: 10.1111/j.1469-8749.1998.tb12324.x

For peer review only - http://bmjopen.bmj.com/site/about/guidelines.xhtml

12. Abel K, Heuvelman H, Wicks S, et al. Gestational age at birth and academic performance: population-based cohort study. *International journal of epidemiology* 2017;46(1):324-35. doi: 10.1093/ije/dyw284 [published Online First: 2016/11/08]
13. European Medicines Agency. Guideline on Missing Data in Confirmatory Clinical Trials 2010.
14. de Jong C, Kikkert HK, Fidler V, et al. Effects of long-chain polyunsaturated fatty acid supplementation of infant formula on cognition and behaviour at 9 years of age. *Developmental medicine and child neurology* 2012;54(12):1102-8. doi: 10.1111/j.1469-8749.2012.04444.x [published Online First: 2012/10/17]
15. Stata Statistical Software: Release 16. [program]. College Station, TX: StataCorp LLC, 2019.
16. Victora CG, Horta BL, de Mola CL, et al. Association between breastfeeding and intelligence, educational attainment, and income at 30 years of age: a prospective birth cohort study from Brazil. *The Lancet Global Health* 2015;3(4):e199-e205. doi: 10.1016/S2214-109X(15)70002-1
17. Lucas A, Morley R, Cole TJ, et al. Breast milk and subsequent intelligence quotient in children born preterm. *Lancet (London, England)* 1992;339(8788):261-4. doi: 10.1016/0140-6736(92)91329-7 [published Online First: 1992/02/01]
18. Fryer Jr RG. The production of human capital in developed countries: Evidence from 196 randomized field experiments. Handbook of economic field experiments: Elsevier 2017:95-322.
19. Kraft MA. Interpreting Effect Sizes of Education Interventions. *Educational Researcher*
20. Higgins JP, Altman DG, Gøtzsche PC, et al. The Cochrane Collaboration's tool for assessing risk of bias in randomised trials. *Bmj* 2011;343:d5928.
21. Fewtrell MS, Kennedy K, Singhal A, et al. How much loss to follow-up is acceptable in long-term randomised trials and prospective studies? *Arch Dis Child* 2008;93(6):458-61. doi: 10.1136/adc.2007.127316 [published Online First: 2008/05/23]
22. Department for Education. Freedom of Information Request by Jen Persson 2017 [Available from: https://www.whatdotheyknow.com/request/pupil_data_national_pupil_databa_3?unfold=1 accessed 28 Mar 2020.
23. Harron K, Dibben C, Boyd J, et al. Challenges in administrative data linkage for research. *Big Data & Society* 2017;4(2):2053951717745678. doi: 10.1177/2053951717745678

For peer review only - http://bmjopen.bmj.com/site/about/guidelines.xhtml

24. Harron K, Wade A, Gilbert R, et al. Evaluating bias due to data linkage error in electronic healthcare records. *BMC Medical Research Methodology* 2014;14(1):36. doi: 10.1186/1471-2288-14-36

25. Harron KL, Doidge JC, Knight HE, et al. A guide to evaluating linkage quality for the analysis of linked data. *International journal of epidemiology* 2017;46(5):1699-710. doi: 10.1093/ije/dyx177 [published Online First: 2017/10/13]

26. Harron K, Wade A, Muller-Pebody B, et al. Opening the black box of record linkage. *J Epidemiol Community Health* 2012;66(12):1198. doi: 10.1136/jech-2012-201376 [published Online First: 2012/06/19]

27. Fellegi IP, Sunter AB. A Theory for Record Linkage. *Journal of the American Statistical Association* 1969;64(328):1183-210. doi: 10.1080/01621459.1969.10501049

28. Barker DJ, Osmond C. Infant mortality, childhood nutrition, and ischaemic heart disease in England and Wales. *The Lancet* 1986;327(8489):1077-81.

29. Lucas A. Long-term programming effects of early nutrition -- implications for the preterm infant. *J Perinatol* 2005;25 Suppl 2:S2-6. doi: 10.1038/sj.jp.7211308 [published Online First: 2005/04/30]

30. UCL Great Ormond Street Institute of Child Health. Information for participants 2020 [Available from: https://www.ucl.ac.uk/child-health/research/population-policy-and-practice/research/studies/head-or-heart-study.

Figure legends/ captions

Figure 1: Combined flow of randomised participants from seven infant formula trials

Figure 2: Data flows

(1) Participant identifier information securely transferred for linkage to FFT. (2) Deterministic linkage at FFT, application of agreement flags to all candidate pairs, and candidate pairs securely sent to UCL minus participant identifiers. (3) Implausible pairs discarded using RCT clinical variables (linked through unique study ID number). (4) Probabilistic linkage at UCL. (5) Data cleaning. UCL University College London; RCT randomised controlled trial; NPD National pupil data base, FFT Fischer-Family Trust.

Figure 3: Trial interventions, comparators and populations

For peer review only - http://bmjopen.bmj.com/site/about/guidelines.xhtml

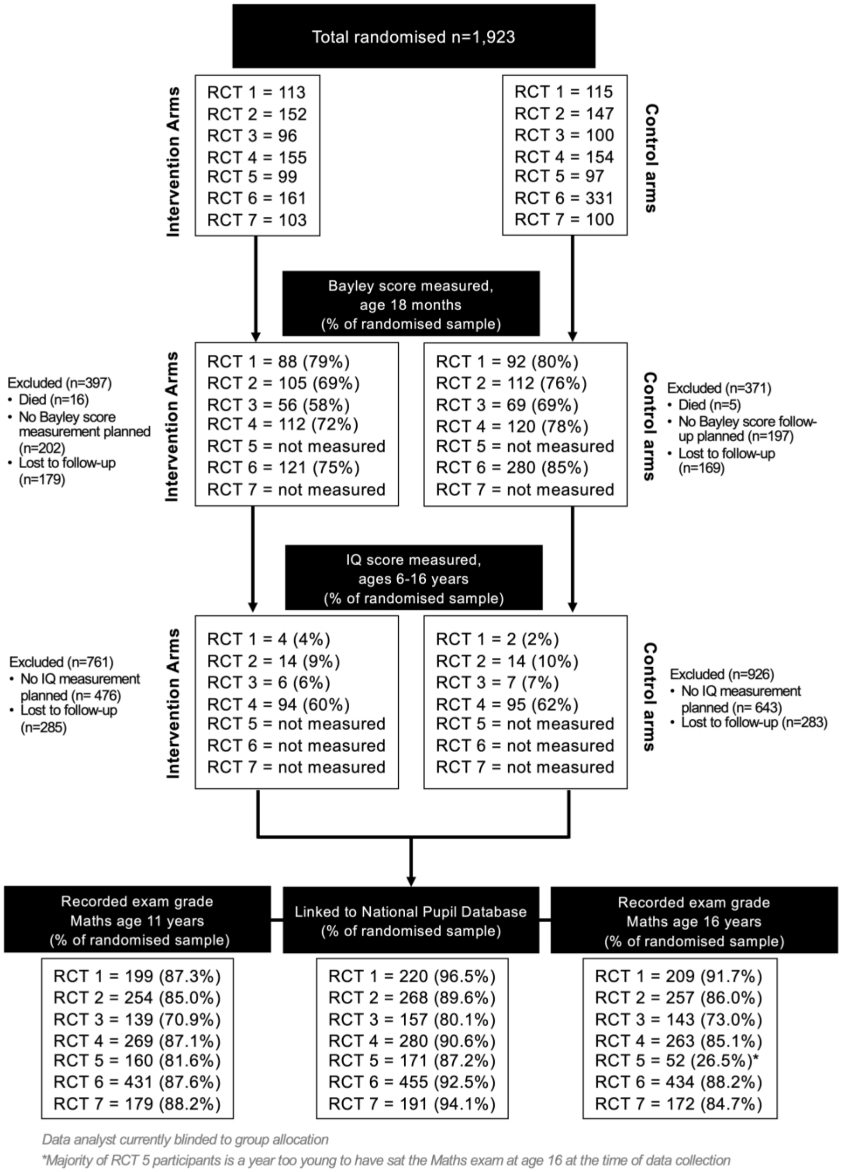

Figure 1 Combined flow of participants of seven infant formula trials

126x175mm (600 x 600 DPI)

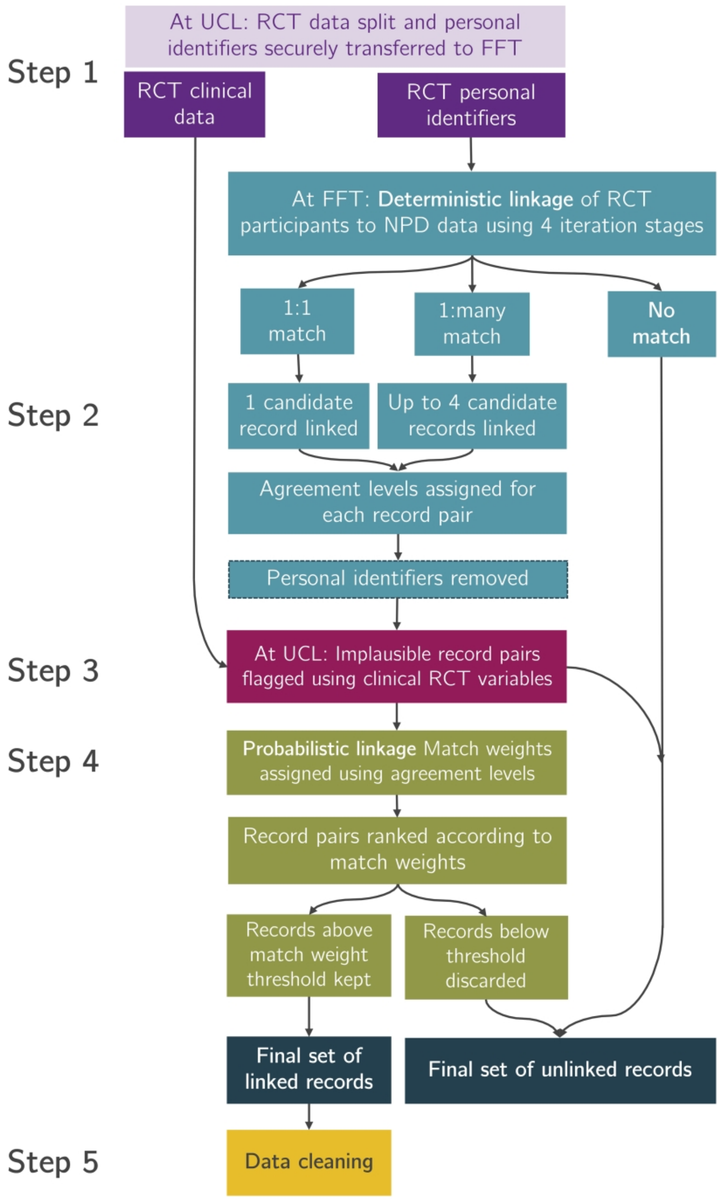

Figure 2 Data Flows. (1) Participant identifier information securely transferred for linkage to FFT. (2) Deterministic linkage at FFT, application of agreement flags to all candidate pairs, and candidate pairs securely sent to UCL minus participant identifiers. (3) Implausible pairs discarded using RCT clinical variables (linked through unique study ID number). (4) Probabilistic linkage at UCL. (5) Data cleaning. UCL University College London; RCT randomised controlled trial; NPD National pupil data base, FFT Fischer-Family Trust.

127x208mm (600 x 600 DPI)

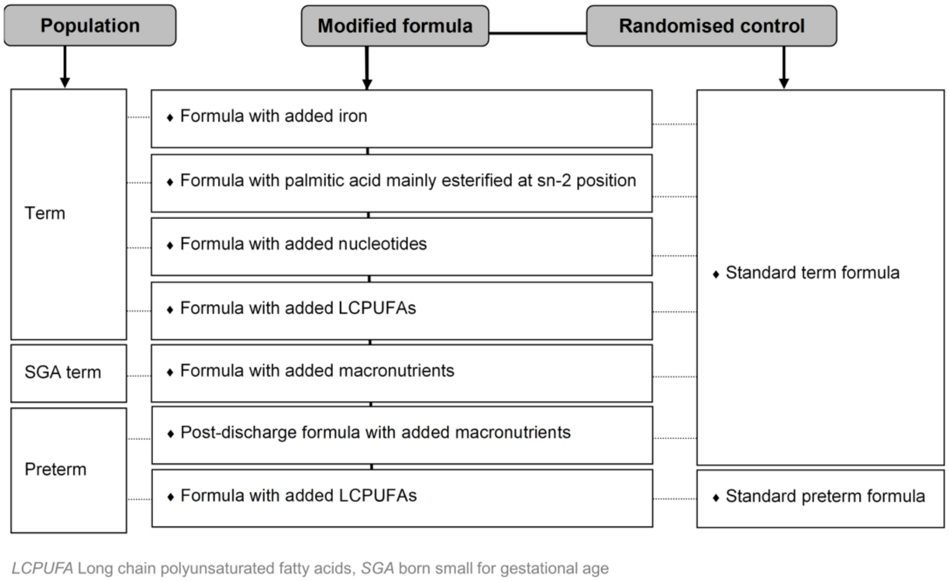

Figure 3 Trial interventions, comparators and population

351x230mm (300 x 300 DPI)

bmjopen-2019-035968 Supplementary material

Supplementary Table 1 Trial Characteristics

	RCT 1	RCT 2	RCT 3	RCT 4	RCT 5	RCT 6	RCT 7
Intervention	Nutrient-enriched	Nutrient-enriched	LCPUFA	LCPUFA	Nucleotides	Iron	Sn-2 palmitate
Population	Preterm	SGA Term	Preterm	Term	Term	Term	Term
Years randomised	1993-96	1993-96	1993-96	1993-95	2000-02	1993-94	1995-96
Eligibility	<1750g bw <37w ga	bw <10th centile ≥37w ga	≤1750g bw ≤37w ga	≥37w ga	≥37w ga	bw > 2500g, ≥37w ga age: 9m	≥37w ga, bw>5th centile
Control formula:	Term formula	Term formula	Preterm formula	Term formula	Term formula	Term formula or cow's milk	Term formula
Intervention: Control formula with added:	Macro nutrients and vitamins	Macro nutrients and vitamins	DHA and AA	DHA and AA	Nucleotides	12 mg/L iron	Sn-2 palmitate
Timing of intervention	1w < discharge - 9m	Birth – 12w	Birth – 3w	Birth – 6m	Birth – 20w	9m - 18m	Birth – 12w
Centre(s)	Nottingham Leicester Cambridge Ipswich	Nottingham Leicester Cambridge	Nottingham Leicester	Nottingham Leicester	Nottingham Leicester	Nottingham Leicester Norwich	Cambridge
Total N	296	475	284	447	296	492	323
Intervention	113	152	96	155	99	161	103
Control (1)	115	147	100	154	97	165	100
Control (2)	-	-	-	-	-	166	-
Non-randomised breastfed reference	68	176	88	138	100	-	120
References	[1]	[2,3]	[4,5]	[6,7]	[8,9]	[10,11]	[12,13]

bw=birthweight, ga=gestational age, d=days, w=week(s), m=moths, LCPUFA=Long Chain Polyunsaturated Fatty Acids, SGA=small-for-gestational-age, m=months,

For peer review only

bmjopen-2019-035968 Supplementary material

Data cleaning methods:

The party who conducts the linkage (the Fischer Family Trust, FFT) provides up to three likely matching pupil IDs and their school records per participant. Only one of these records can be a true match and it is possible that none of the linked pupil IDs is a true match, as participants might have emigrated, died or attended private schools which do not transmit data to the national pupil database. We will check and cross validate the data provided by FFT extensively, using auxiliary variables from the trials (such as known death) and linkage confidence flags supplied by FFT (Supplementary Table 2).

Supplementary Table 2 Possible match levels

Identifier	Description
First name	First name and other first name both exact match
	First name matches other name in both directions
	First name exact match
	First name matches other name
	Other name exact match
	First name truncated at any hyphen matches
	First name matches via common name alternatives lookup
	Pattern match function
	Pattern match function - AND first character of first name matches
	First name / surname match in both directions
	No Link
Surname	Surname exact match (including alternative surnames)
	Surname truncated at any hyphen matches
	Pattern match function
	Pattern match function - AND first character of surname matches
	First name / surname match in both directions
	No Link
Date of Birth	DOB exact match
	Day on source matches month on match, and vice versa; year matches (i.e. transposed date)
	Day and month match (i.e. wrong year)
	Day and year match (i.e. wrong month)
	Month and year match (i.e. wrong day)
	Either source or match DOB is 1st January; year matches
	Either source or match DOB is 1st September; year matches
	No Link
Location	Postcode exact match
	Local Authority match
	Neighbouring / nearby Local Authority match

For peer review only - http://bmjopen.bmj.com/site/about/guidelines.xhtml

bmjopen-2019-035968 Supplementary material

No Link

We will calculate match-weights for each participant-pupil pair according to a probabilistic method adapted from Fellegi and Sunter.[14] These match-weights will then be used to rank the pairs according to their probability of being a true match. For each match category (Supplementary Table 2) we will derive proxy-m and proxy-u probabilities which will then be used to produce the match weight.

M-probability: probability of agreeing on a category given that it is a true match
U-probability: probability of agreeing on a category given by chance

The original method requires a set of participant-pupil pairs that are certain true matches and a set of participant-pupil pairs that are certain false matches. Due to the de-identified nature of the data, such information will not be available and thus needs to be approximated. True matches will be defined as participants perfectly matching all categories (except the one we are calculating the probability for). Certain false matches will be defined as participants disagreeing in all categories (except the one we are calculating the probability for). This will yield a total of eight reference datasets. For each linkage flag (sex, first name, last name, date of birth, location) we will then calculate the variable weight using:

$$\log2\ (m/u)$$

The final match weight for a participant-pupil pair will be calculated using the sum of all weights in a pair:

$$\sum \log2\ (m/u)$$

At this stage each participant will still have up to three multiple possible matches.
First, we will drop all participants that linked but where their age at first link was >17 and they do not agree with the record on date of birth. This is because the date of birth in the trial data is very likely to be correct as it corresponds to the recruitment periods and was confirmed repeatedly during the follow-up period. We will also discard all records for children that died before school age (4 years) where information on death and time of death during the trial period is available.

We will then rank participant-pupil pairs according to their match weights. If the highest match-weight for a participant is 10% above that of the second best match, we will automatically keep the best match and discard the other(s). If the difference is lower, we will manually review these

For peer review only - http://bmjopen.bmj.com/site/about/guidelines.xhtml

records to decide which one is the best match (if any). Decisions taken at manual review will be reported to ensure reproducibility. We will then choose two cut-offs based on visual inspection of the match probabilities; one threshold to maximise sensitivity (many true positive links) and one to maximise specificity (few false positive links). To determine how representative each sample defined by these cut-offs is of the original randomised cohort, we will compare them across the variables that were measured at randomisation. We will use the bigger, more sensitive sample in our analyses but use the smaller, more specific sample in sensitivity analyses.

For peer review only

bmjopen-2019-035968 Supplementary material

Trial formula compositions:

RCT 1: Preterm nutrient enriched formula (post-discharge) vs standard formula

per 100 ml	Standard formula	Modified formula
Energy (kcal)	68	72
Protein (g)	1.45	1.85
Casein	0.56	0.72
Whey	0.89	1.13
Carbohydrate (g)	6.96	7.24
Lactose (g)	6.96	6.20
Maltodextrin (g)	-	1.04
Fat (g)	3.82	3.96
Minerals		
Calcium (mg)	39	70
Phosphorus (mg)	27	35
Sodium (mg)	17	22
Chloride (mg)	45	45
Potassium (mg)	57	78
Iron (mg)	0.65	0.65
Zinc (mg)	0.34	0.60
Copper (µg)	42	57
Iodine (µg)	4.5	4.5
Magnesium (mg)	5.2	5.2
Manganese (µg)	3.4	5
Vitamins		
Vitamin A (µg)	100	100
Thiamine B_1 (mg)	42	95
Riboflavin B_2 (mg)	55	100
Niacin B_3 (µg)		
Pantothenic acid B_5 (mg)	0.23	0.40
Pyridoxine B_6 (µg)	35	80
Biotin B_7 (µg)	1.0	1.1
Inositol B_8 (mg)		
Folate B_9 (µg)	3.4	25
Cyanocobalamin B_{12} (µg)	0.14	0.2
Vitamin C (mg)	6.9	15
Vitamin D (µg)	1.0	1.3
Vitamin E (mg)	0.48	1.5
Vitamin K (µg)	2.7	6.0
Choline (mg)	4.8	5.1
Taurine (mg)	5	5.1
Carnitine (mg)	-	1.1

For peer review only

For peer review only - http://bmjopen.bmj.com/site/about/guidelines.xhtml

bmjopen-2019-035968 Supplementary material

RCT 2: Term SGA nutrient enriched formula vs standard formula

per 100 ml	Standard formula	Modified formula
Energy (kcal)	68	72
Protein (g)	1.45	1.85
Casein	0.56	0.72
Whey	0.89	1.13
Carbohydrate (g)	6.96	7.24
Lactose (g)	6.96	6.20
Maltodextrin (g)	-	1.04
Fat (g)	3.82	3.96
Minerals		
Calcium (mg)	39	70
Phosphorus (mg)	27	35
Sodium (mg)	17	22
Chloride (mg)	45	45
Potassium (mg)	57	78
Iron (mg)	0.65	0.65
Zinc (mg)	0.34	0.60
Copper (µg)	42	57
Iodine (µg)	4.5	4.5
Magnesium (mg)	5.2	5.2
Manganese (µg)	3.4	5
Vitamins		
Vitamin A (µg)	100	100
Thiamine B_1 (mg)	42	95
Riboflavin B_2 (mg)	55	100
Niacin B_3 (µg)		
Pantothenic acid B_5 (mg)	0.23	0.40
Pyridoxine B_6 (µg)	35	80
Biotin B_7 (µg)	1.0	1.1
Inositol B_8 (mg)		
Folate B_9 (µg)	3.4	25
Cyanocobalamin B_{12} (µg)	0.14	0.2
Vitamin C (mg)	6.9	15
Vitamin D (µg)	1.0	1.3
Vitamin E (mg)	0.48	1.5
Vitamin K (µg)	2.7	6.0
Choline (mg)	4.8	5.1
Taurine (mg)	5.0	5.1
Carnitine (mg)	-	1.1

RCT 3: Preterm LCPUFA-enriched formula vs standard preterm formula

per 100 ml	Standard formula	Modified formula
Energy (kcal)	70	70

For peer review only

bmjopen-2019-035968 Supplementary material

Protein (g)	2.0	2.0
Casein	0.8	0.8
Whey	1.2	1.2
Carbohydrate (g)	7.7	7.7
Total fat (g)	3.5	3.5
Fatty acid composition (g/ 100 g fat)		
C8:0 caprylic	0.7	0.6
C10:0 capric	1.2	1.1
C12:0 lauric	6.3	4.9
C14:0 myristic	5.6	5.6
C16:0 palmitic	25.8	26.3
C18:0 stearic	8.2	8.5
C18:1 oleic	32.6	32.9
C18:2 n-6 linoleic	10.6	12.0
C18:3 n-6 γ-linolenic	0.1	0.4
C18:3 n-6 α-linolenic	0.7	0.6
C20:0 arachidic	0.4	0.3
C20:1 n-9 eicosanoic	0.2	0.3
C20:4 n-6 AA	-	0.31
C20:5 n-3 eicosapentaenoic	-	0.04
C22:0	0.3	0.2
C22:6 n-3 DHA	-	0.17
C24:0	0.2	0.1
Other fatty acids	7.1	4.62
Cholesterol	-	7.73
Minerals		
Calcium (mg)	70	70
Phosphorus (mg)	35	42
Sodium (mg)	30	27
Potassium (mg)	75	71
Iron (mg)	0.1	0.07
Zinc (mg)	0.39	0.4
Vitamins		
Vitamin A (μg)	63	63
Vitamin D (μg)	2.1	2.1
Vitamin E (mg)	2.0	2.0
Vitamin K (μg)	2.8	3.0

RCT 4: Term LCPUFA-enriched formula vs standard term formula

per 100 ml	Standard formula	Modified formula
Energy (kcal)	67	67
Protein (g) (casein:whey 40:60)	1.5	1.5
Carbohydrate (g)	7.6	7.7
Lactose (g)	7.6	7.7
Total fat (g)	3.4	3.4
Fatty acid composition (% total fat)		
C8:0 caprylic	0.8	2.0
C10:0 capric	2.4	2.0

For peer review only

C12:0 lauric	1.6	12.3
C14:0 myristic	8.6	5.2
C16:0 palmitic	23.3	25.6
C18:0 stearic	10.2	4.6
C18:1 oleic	32.7	29.7
C18:2 n-6 linoleic	12.4	15.9
C18:3 n-6 α-linolenic	1.1	1.4
C20:4 n-6 AA	-	0.30
C20:5 n-3 eicosapentaenoic	-	0.01
C22:6 n-3 DHA	-	0.32
Cholesterol	<0.5	0.8
Minerals		
Iron (mg)	0.8	0.8

RCT 5: Nucleotide-enriched formula vs standard formula

per 100 ml	Standard formula	Modified formula
Energy (kcal)	68	68
Protein (g) (casein:whey 40:60)	1.5	1.5
Casein	0.6	0.6
Whey	0.9	0.9
Carbohydrate as lactose (g)	7.0	7.0
Total fat (g)	3.8	3.8
C18:2 n-6 linoleic (mg)	350	350
C18:3 n-6 γ-linolenic (mg)	33	33
C18:3 n-6 α-linolenic (mg)	44	44
LCPUFAs (mg)	26	26
Nucleotides		
Cytidine monophosphate (mg)	0.3	1.5
Uridine monophosphate (mg)	-	0.5
Adenosine monophosphate (mg)	-	0.6
Guanosine monophosphate (mg)	-	0.2
Inosine monophosphate (mg)	-	0.3
Minerals		
Calcium (mg)	39	39
Magnesium (mg)	5.2	5.2
Manganese (µg)	3.4	3.4
Phosphorus (mg)	27	27
Sodium (mg)	17	17
Potassium (mg)	0.3	0.3
Iron (mg)	0.6	0.6
Zinc (mg)	0.3	0.3
Copper (µg)	42	42
Iodine (µg)	4.5	4.5
Vitamins		
Vitamin A (µg)	100	100
Thiamine B_1 (mg)	42	42
Riboflavin B_2 (mg)	55	55

For peer review only - http://bmjopen.bmj.com/site/about/guidelines.xhtml

Niacin B_3 (mg)	0.7	0.7
Pantothenic acid B_5 (µg)	0.2	0.2
Biotin B_7 (µg)	1.0	1.0
Vitamin B_8 (µg)	35	35
Folic Acid B_9 (µg)	3.4	3.4
Cyanocobalamin B_{12} (µg)	0.14	0.14
Vitamin C (mg)	6.9	6.9
Vitamin D (µg)	1.0	1.0
Vitamin E (mg)	0.5	0.5
Vitamin K (µg)	2.7	2.7
Choline (mg)	4.8	4.8
Taurine (mg)	5.0	5.0

RCT 6: Iron-enriched formula vs standard formula

per 100 ml	Standard formula	Modified formula
Energy (kcal)	65	65
Protein (g)	2.5	2.5
Carbohydrate (g)	8.0	8.0
Lactose (g)	6.4	6.4
Maltodextrin	1.6	1.6
Fat (g)	2.8	2.8
Saturated (%)	42.9	42.9
Unsaturated (%)	57.1	57.1
Minerals		
Calcium (mg)	100	100
Phosphorus (mg)	65	65
Magnesium (mg)	7.5	7.5
Sodium (mg)	30	30
Potassium (mg)	100	100
Chloride (mg)	70	70
Iron (mg)	0.9	1.2
Zinc (mg)	0.5	0.5
Iodine (µg)	6.9	6.9
Vitamins		
Vitamin A (µg)	6.9	6.9
Thiamine B_1 (mg)	0.8	0.8
Riboflavin B_2 (mg)	0.12	0.12
Niacin B_3 (µg)	0.61	0.61
Pantothenic acid B_5 (µg)	0.24	0.24
Pyridoxine B_6 (µg)	48	48
Biotin B_7 (µg)	1.71	1.71
Inositol B_8 (mg)		
Folate B_9 (µg)	6.0	6.0
Cyanocobalamin B_{12} (µg)	0.12	0.12

For peer review only - http://bmjopen.bmj.com/site/about/guidelines.xhtml

bmjopen-2019-035968 Supplementary material

Vitamin C (µg)	6.6	6.6
Vitamin D (µg)	1.2	1.2
Vitamin E (mg)	0.75	0.75
Vitamin K (µg)	6.6	6.6

RCT 7: sn-2 Palmitate enriched formula vs standard formula

per 100 ml	Standard formula	Modified formula
Energy (kcal)	70	73
Protein (g)	1.6	1.6
Carbohydrate (g)	7.1	7.1
Lactose (g)	7.1	7.1
Total fat (g)	3.9	4.2
Fatty acid composition (% total fat)		
C8:0 caprylic	1.6	1.5
C10:0 capric	0.5	0.4
C12:0 lauric	10.3	12.4
C14:0 myristic	4.9	4.7
C16:0 palmitic	19.6 (12% in sn-2)	20.1 (50% in sn-2)
C18:0 stearic	3.9	3.1
C18:1 n-9 oleic	41.3	42
C18:2 n-6 linoleic	12.1	13
C18:3 n-3 α-linolenic	1.9	1.6
Other fatty acids	4.0	1.2
Minerals		
Calcium (mg)	54	57
Phosphorus (mg)	32	33
Sodium (mg)	23	23
Potassium (mg)	76	74
Chloride (mg)	45	44
Magnesium (mg)	6.3	6.3
Manganese (µg)	5.1	5.2
Iron (mg)	0.51	0.57
Zinc (mg)	0.45	0.49
Copper (µg)	44	45
Iodine (µg)	10	10
Vitamins		
Vitamin A (µg)	92	95
Thiamine B_1 (µg)	40	40
Riboflavin B_2 (µg)	100	100
Pyridoxine B_6 (µg)	40	40
Vitamin D (µg)	1.1	1.1
Vitamin K (µg)	5	5
Vitamin E (mg)	1.1	1.1

For peer review only

References:

1. Lucas A, Fewtrell MS, Morley R, et al. Randomized trial of nutrient-enriched formula versus standard formula for postdischarge preterm infants. Pediatrics 2001;108(3):703-11. [published Online First: 2001/09/05]
2. Fewtrell MS, Morley R, Abbott RA, et al. Catch-up growth in small-for-gestational-age term infants: a randomized trial. The American journal of clinical nutrition 2001;74(4):516-23. doi: 10.1093/ajcn/74.4.516 [published Online First: 2001/09/22]
3. Morley R, Fewtrell MS, Abbott RA, et al. Neurodevelopment in children born small for gestational age: a randomized trial of nutrient-enriched versus standard formula and comparison with a reference breastfed group. Pediatrics 2004;113(3 Pt 1):515-21. [published Online First: 2004/03/03]
4. Fewtrell MS, Morley R, Abbott RA, et al. Double-Blind, Randomized Trial of Long-Chain Polyunsaturated Fatty Acid Supplementation in Formula Fed to Preterm Infants. Pediatrics 2002;110(1):73-82. doi: 10.1542/peds.110.1.73
5. Fewtrell MS, Abbott RA, Kennedy K, et al. Randomized, double-blind trial of long-chain polyunsaturated fatty acid supplementation with fish oil and borage oil in preterm infants. The Journal of pediatrics 2004;144(4):471-9. doi: 10.1016/j.jpeds.2004.01.034 [published Online First: 2004/04/08]
6. Lucas A, Stafford M, Morley R, et al. Efficacy and safety of long-chain polyunsaturated fatty acid supplementation of infant-formula milk: a randomised trial. Lancet (London, England) 1999;354(9194):1948-54. doi: 10.1016/s0140-6736(99)02314-4 [published Online First: 2000/01/06]
7. Singhal A, Morley R, Cole TJ, et al. Infant nutrition and stereoacuity at age 4-6 y. The American journal of clinical nutrition 2007;85(1):152-9. doi: 10.1093/ajcn/85.1.152 [published Online First: 2007/01/09]
8. Singhal A, Kennedy K, Lanigan J, et al. Dietary Nucleotides and Early Growth in Formula-Fed Infants: A Randomized Controlled Trial. Pediatrics 2010;126(4):e946-e53. doi: 10.1542/peds.2009-2609
9. Singhal A, Macfarlane G, Macfarlane S, et al. Dietary nucleotides and fecal microbiota in formula-fed infants: a randomized controlled trial. The American journal of clinical nutrition 2008;87(6):1785-92. [published Online First: 2008/06/11]
10. Morley R, Abbott R, Fairweather-Tait S, et al. Iron fortified follow on formula from 9 to 18 months improves iron status but not development or growth: a randomised trial. Archives of Disease in Childhood 1999;81(3):247-52. doi: 10.1136/adc.81.3.247
11. Singhal A, Morley R, Abbott R, et al. Clinical safety of iron-fortified formulas. Pediatrics 2000;105(3):E38. doi: 10.1542/peds.105.3.e38 [published Online First: 2000/03/04]
12. Kennedy K, Fewtrell MS, Morley R, et al. Double-blind, randomized trial of a synthetic triacylglycerol in formula-fed term infants: effects on stool biochemistry, stool characteristics, and bone mineralization. The American journal of clinical nutrition 1999;70(5):920-7. doi: 10.1093/ajcn/70.5.920 [published Online First: 1999/10/28]
13. Fewtrell MS, Kennedy K, Murgatroyd PR, et al. Breast-feeding and formula feeding in healthy term infants and bone health at age 10 years. Br J Nutr 2013;110(6):1061-7. doi: 10.1017/s0007114512006149 [published Online First: 2013/02/08]
14. Fellegi IP, Sunter AB. A Theory for Record Linkage. Journal of the American Statistical Association 1969;64(328):1183-210. doi: 10.1080/01621459.1969.10501049

For peer review only

STANDARD PROTOCOL ITEMS: RECOMMENDATIONS FOR INTERVENTIONAL TRIALS

SPIRIT 2013 Checklist: Recommended items to address in a clinical trial protocol and related documents*

Section/item	Item No	Description	Page
**Administrative information**			
Title	1	Descriptive title identifying the study design, population, interventions, and, if applicable, trial acronym	1
Trial registration	2a	Trial identifier and registry name. If not yet registered, name of intended registry	n/a
	2b	All items from the World Health Organization Trial Registration Data Set	n/a
Protocol version	3	Date and version identifier	all
Funding	4	Sources and types of financial, material, and other support	13
Roles and responsibilities	5a	Names, affiliations, and roles of protocol contributors	1
	5b	Name and contact information for the trial sponsor	1
	5c	Role of study sponsor and funders, if any, in study design; collection, management, analysis, and interpretation of data; writing of the report; and the decision to submit the report for publication, including whether they will have ultimate authority over any of these activities	13
	5d	Composition, roles, and responsibilities of the coordinating centre, steering committee, endpoint adjudication committee, data management team, and other individuals or groups overseeing the trial, if applicable (see Item 21a for data monitoring committee)	13
**Introduction**			
Background and rationale	6a	Description of research question and justification for undertaking the trial, including summary of relevant studies (published and unpublished) examining benefits and harms for each intervention	13
	6b	Explanation for choice of comparators	n/a
Objectives	7	Specific objectives or hypotheses	4-5

For peer review only

Trial design	8	Description of trial design including type of trial (eg, parallel group, crossover, factorial, single group), allocation ratio, and framework (eg, superiority, equivalence, noninferiority, exploratory)	5

Methods: Participants, interventions, and outcomes

Study setting	9	Description of study settings (eg, community clinic, academic hospital) and list of countries where data will be collected. Reference to where list of study sites can be obtained	Supplementary material Table 1
Eligibility criteria	10	Inclusion and exclusion criteria for participants. If applicable, eligibility criteria for study centres and individuals who will perform the interventions (eg, surgeons, psychotherapists)	Supplementary material Table 1
Interventions	11a	Interventions for each group with sufficient detail to allow replication, including how and when they will be administered	Supplementary material Compositions
	11b	Criteria for discontinuing or modifying allocated interventions for a given trial participant (eg, drug dose change in response to harms, participant request, or improving/worsening disease)	n/a
	11c	Strategies to improve adherence to intervention protocols, and any procedures for monitoring adherence (eg, drug tablet return, laboratory tests)	Supplementary material
	11d	Relevant concomitant care and interventions that are permitted or prohibited during the trial	n/a

For peer review only - http://bmjopen.bmj.com/site/about/guidelines.xhtml

Outcomes	12	Primary, secondary, and other outcomes, including the specific measurement variable (eg, systolic blood pressure), analysis metric (eg, change from baseline, final value, time to event), method of aggregation (eg, median, proportion), and time point for each outcome. Explanation of the clinical relevance of chosen efficacy and harm outcomes is strongly recommended	6
Participant timeline	13	Time schedule of enrolment, interventions (including any run-ins and washouts), assessments, and visits for participants. A schematic diagram is highly recommended (see Figure)	Figure 1
Sample size	14	Estimated number of participants needed to achieve study objectives and how it was determined, including clinical and statistical assumptions supporting any sample size calculations	10
Recruitment	15	Strategies for achieving adequate participant enrolment to reach target sample size	Supplementary material

Methods: Assignment of interventions (for controlled trials)

Allocation:

Sequence generation	16a	Method of generating the allocation sequence (eg, computer-generated random numbers), and list of any factors for stratification. To reduce predictability of a random sequence, details of any planned restriction (eg, blocking) should be provided in a separate document that is unavailable to those who enrol participants or assign interventions	n/a
Allocation concealment mechanism	16b	Mechanism of implementing the allocation sequence (eg, central telephone; sequentially numbered, opaque, sealed envelopes), describing any steps to conceal the sequence until interventions are assigned	n/a
Implementation	16c	Who will generate the allocation sequence, who will enrol participants, and who will assign participants to interventions	n/a
Blinding (masking)	17a	Who will be blinded after assignment to interventions (eg, trial participants, care providers, outcome assessors, data analysts), and how	n/a

	17b	If blinded, circumstances under which unblinding is permissible, and procedure for revealing a participant's allocated intervention during the trial	n/a
**Methods: Data collection, management, and analysis**			
Data collection methods	18a	Plans for assessment and collection of outcome, baseline, and other trial data, including any related processes to promote data quality (eg, duplicate measurements, training of assessors) and a description of study instruments (eg, questionnaires, laboratory tests) along with their reliability and validity, if known. Reference to where data collection forms can be found, if not in the protocol	5
	18b	Plans to promote participant retention and complete follow-up, including list of any outcome data to be collected for participants who discontinue or deviate from intervention protocols	5
Data management	19	Plans for data entry, coding, security, and storage, including any related processes to promote data quality (eg, double data entry; range checks for data values). Reference to where details of data management procedures can be found, if not in the protocol	Supplementary material
Statistical methods	20a	Statistical methods for analysing primary and secondary outcomes. Reference to where other details of the statistical analysis plan can be found, if not in the protocol	8
	20b	Methods for any additional analyses (eg, subgroup and adjusted analyses)	9
	20c	Definition of analysis population relating to protocol non-adherence (eg, as randomised analysis), and any statistical methods to handle missing data (eg, multiple imputation)	10
**Methods: Monitoring**			
Data monitoring	21a	Composition of data monitoring committee (DMC); summary of its role and reporting structure; statement of whether it is independent from the sponsor and competing interests; and reference to where further details about its charter can be found, if not in the protocol. Alternatively, an explanation of why a DMC is not needed	n/a
	21b	Description of any interim analyses and stopping guidelines, including who will have access to these interim results and make the final decision to terminate the trial	n/a

For peer review only - http://bmjopen.bmj.com/site/about/guidelines.xhtml

Harms	22	Plans for collecting, assessing, reporting, and managing solicited and spontaneously reported adverse events and other unintended effects of trial interventions or trial conduct	n/a
Auditing	23	Frequency and procedures for auditing trial conduct, if any, and whether the process will be independent from investigators and the sponsor	n/a
**Ethics and dissemination**			
Research ethics approval	24	Plans for seeking research ethics committee/institutional review board (REC/IRB) approval	12
Protocol amendments	25	Plans for communicating important protocol modifications (eg, changes to eligibility criteria, outcomes, analyses) to relevant parties (eg, investigators, REC/IRBs, trial participants, trial registries, journals, regulators)	12
Consent or assent	26a	Who will obtain informed consent or assent from potential trial participants or authorised surrogates, and how (see Item 32)	12
	26b	Additional consent provisions for collection and use of participant data and biological specimens in ancillary studies, if applicable	12
Confidentiality	27	How personal information about potential and enrolled participants will be collected, shared, and maintained in order to protect confidentiality before, during, and after the trial	12-13
Declaration of interests	28	Financial and other competing interests for principal investigators for the overall trial and each study site	14
Access to data	29	Statement of who will have access to the final trial dataset, and disclosure of contractual agreements that limit such access for investigators	14
Ancillary and post-trial care	30	Provisions, if any, for ancillary and post-trial care, and for compensation to those who suffer harm from trial participation	n/a
Dissemination policy	31a	Plans for investigators and sponsor to communicate trial results to participants, healthcare professionals, the public, and other relevant groups (eg, via publication, reporting in results databases, or other data sharing arrangements), including any publication restrictions	13
	31b	Authorship eligibility guidelines and any intended use of professional writers	n/a

For peer review only - http://bmjopen.bmj.com/site/about/guidelines.xhtml

	31c	Plans, if any, for granting public access to the full protocol, participant-level dataset, and statistical code	14
**Appendices**			
Informed consent materials	32	Model consent form and other related documentation given to participants and authorised surrogates	n/a
Biological specimens	33	Plans for collection, laboratory evaluation, and storage of biological specimens for genetic or molecular analysis in the current trial and for future use in ancillary studies, if applicable	n/a

*It is strongly recommended that this checklist be read in conjunction with the SPIRIT 2013 Explanation & Elaboration for important clarification on the items. Amendments to the protocol should be tracked and dated. The SPIRIT checklist is copyrighted by the SPIRIT Group under the Creative Commons "Attribution-NonCommercial-NoDerivs 3.0 Unported" license.

For peer review only

BMJ Open

Infant formula composition and educational performance: a protocol to extend follow-up for a set of randomised controlled trials using linked administrative education records

Journal:	*BMJ Open*
Manuscript ID	bmjopen-2019-035968.R2
Article Type:	Protocol
Date Submitted by the Author:	03-Jun-2020
Complete List of Authors:	Verfürden, Maximiliane ; University College London, Great Ormond Street Institute of Child Health<br>Harron, Katie; University College London Institute of Child Health,<br>Jerrim, John; University College London, Institute of Education<br>Fewtrell, Mary; Great Ormond Street Hospital for Children,<br>Gilbert, Ruth; UCL Institute of Child Health, Centre for Paediatric Epidemiology and Biostatistics
**Primary Subject Heading**:	Nutrition and metabolism
Secondary Subject Heading:	Health informatics, Paediatrics, Public health
Keywords:	Paediatric neurology < PAEDIATRICS, PUBLIC HEALTH, Clinical trials < THERAPEUTICS, NUTRITION & DIETETICS

SCHOLARONE™
Manuscripts

I, the Submitting Author has the right to grant and does grant on behalf of all authors of the Work (as defined in the below author licence), an exclusive licence and/or a non-exclusive licence for contributions from authors who are: i) UK Crown employees; ii) where BMJ has agreed a CC-BY licence shall apply, and/or iii) in accordance with the terms applicable for US Federal Government officers or employees acting as part of their official duties; on a worldwide, perpetual, irrevocable, royalty-free basis to BMJ Publishing Group Ltd ("BMJ") its licensees and where the relevant Journal is co-owned by BMJ to the co-owners of the Journal, to publish the Work in this journal and any other BMJ products and to exploit all rights, as set out in our licence.

The Submitting Author accepts and understands that any supply made under these terms is made by BMJ to the Submitting Author unless you are acting as an employee on behalf of your employer or a postgraduate student of an affiliated institution which is paying any applicable article publishing charge ("APC") for Open Access articles. Where the Submitting Author wishes to make the Work available on an Open Access basis (and intends to pay the relevant APC), the terms of reuse of such Open Access shall be governed by a Creative Commons licence – details of these licences and which Creative Commons licence will apply to this Work are set out in our licence referred to above.

Other than as permitted in any relevant BMJ Author's Self Archiving Policies, I confirm this Work has not been accepted for publication elsewhere, is not being considered for publication elsewhere and does not duplicate material already published. I confirm all authors consent to publication of this Work and authorise the granting of this licence.

For peer review only

Protocol bmjopen-2019-035968 R.1

Infant formula composition and educational performance: a protocol to extend follow-up for a set of randomised controlled trials using linked administrative education records

Authors:

Maximiliane L. Verfürden, *PhD student*[1] (m.verfuerden@ucl.ac.uk) *
Katie Harron, *Non-Clinical Senior Lecturer*[1,3] (k.harron@ucl.ac.uk)
John Jerrim, *Professor of Education and Social Statistics*[2] (j.jerrim@ucl.ac.uk)
Mary Fewtrell, *Professor of Paediatric Nutrition*[1] (m.fewtrell@ucl.ac.uk)
Ruth E Gilbert, *Professor of Clinical Epidemiology*[1,3] (r.gilbert@ucl.ac.uk)

[1]University College London Great Ormond Street Institute of Child Health
[2]University College London Institute of Education
[3]Health Data Research UK, London, United Kingdom

Correspondence to:
Maximiliane L. Verfuerden
Population, Policy and Practice Research & Teaching Department
UCL Great Ormond Street Institute of Child Health
30 Guilford Street London WC1N 1EH
Tel: 020 7905 2606

Manuscript word count: 3,032

Acknowledgements: The authors thank the participants of the trials and their parents as well as the original investigators for the valuable time they have invested in generating this data. We also thank the peer reviewers for their valuable comments, which in our view have significantly improved the quality of our protocol.

For peer review only - http://bmjopen.bmj.com/site/about/guidelines.xhtml

Abstract

Introduction

The effect of infant nutrition on long-term cognition is important for parents and policy makers. However, most clinical trials typically have short follow-up periods, when measures of cognition are poorly predictive of later function. The few trials with longer-term follow-up have high levels of attrition, which can lead to selection bias, and in turn to erroneous interpretation of long-term harms and benefits of infant nutrition. We address the need for unbiased, long-term follow-up, by linking measures of educational performance from administrative education records. Educational performance is a meaningful marker of cognitive function in children and it is strongly correlated with intelligence quotient. We aim to evaluate educational performance for children who, as infants, were part of a series of trials that randomised participants to either nutritionally modified infant formula or standard formula. Most trialists anticipated positive effects of these interventions on later cognitive function.

Methods and analysis

Using data from 1,923 participants of seven randomised infant formula trials linked to the English National Pupil Database (NPD), this study will provide new insights into the effect of nutrient intake in infancy on school achievement. Our primary outcome will be the mean differences in z-scores between intervention and control groups for a compulsory Mathematics exam sat at age 16. Secondary outcomes will be z-scores for a compulsory English exam at age 16 and z-scores for compulsory Mathematics and English exams at age 11. We will also evaluate intervention effects on the likelihood of receiving Special Educational Needs (SEN) support. All analyses will be performed separately by trial.

Ethics and dissemination

Research ethics approval, and approval from the Health Research Authority Confidentiality Advisory Group, has been obtained for this study. The results of this study will be disseminated to scientific, practitioner and lay audiences, submitted for publication in peer-reviewed journals, and will contribute towards a PhD dissertation.

For peer review only - http://bmjopen.bmj.com/site/about/guidelines.xhtml

Article Summary

Strengths and limitations of this study

- This study uses seven infant formula trials conducted in England to determine whether a range of nutritional interventions during infancy affects educational performance.
- We showcase the research potential of linking extant trials to administrative data, which can offer a low cost way to extend follow-up of early nutrition trials, maintain high rates of follow-up, and safeguard confidentiality.
- There is no agreement on what defines educational performance and at what age it is best measured to determine intervention effects. We therefore rely on data from two objectively assessed, compulsory examinations sat by the majority of state school students in England.

For peer review only

For peer review only - http://bmjopen.bmj.com/site/about/guidelines.xhtml

Introduction

Rationale

There is a need for high quality evidence on the long-term effects of infant formula on cognitive function to inform infant feeding recommendations and guide parents. However, the majority of randomised controlled trials (RCTs) report only short-term effects of formula composition on proxy end-points for cognition such as the Bayley Scales of Infant Development before two years of age.[1] Such early measures are crude, highly observer dependent and have poor predictive properties for later academic and employment outcomes.[2] Better measures such as IQ scores are available, but limited by methodological issues such as small sample sizes, high attrition and no intention-to-treat analyses.[3 4]

Advances in the availability and quality of administrative datasets have created new possibilities for obtaining long-term outcomes on children's educational performance, which is a good predictor of future academic and employment opportunities.[5] Extending extant trial cohorts with educational performance data is achievable where trial data and participant identifiers have been retained and governance arrangements allow secure linkage without the need for participants to re-engage. Educational performance is highly correlated with IQ scores[6] but socially more relevant than IQ scores as it determines future academic and employment opportunities.[7] Linkage to administrative records is available for a fraction of the cost compared to setting up new RCTs or using traditional follow-up methods and could offer a more rapid, complete, and therefore less biased way to determine long-term intervention effects.[8 9]

We sought to determine long-term effects of modified infant formulas by linking RCTs to educational performance data for seven existing trial cohorts that compared modified infant formula interventions with standard infant formula.

Research objective 1

To determine the effect of nutritionally modified infant formula on educational performance in standardised Mathematics and English language tests at age 11 and 16 years.

For peer review only - http://bmjopen.bmj.com/site/about/guidelines.xhtml

Research objective 2

To assess whether infant formula modifications have an effect on the risk of receiving special educational needs support during school.

Research objective 3

To explore whether the effect of infant formula modifications on educational performance differs for boys and girls, by maternal smoking status during pregnancy, birthweight, or maturity at birth.

Methods

Data sources and linkage

We will follow 1,923 participants from seven separate infant formula trials that randomised infants between 1993 and 2002 and were conducted in England (**Figure 1**). Full details on the individual trial cohorts as well as previously reported outcomes can be found elsewhere (**Supplementary Table 1**).

Outcome data for this follow-up will be retrieved from the National Pupil Database (NPD), an administrative data resource containing pupil-level and school-level data on all pupils in state schools in England and held by the Department for Education (DfE).[10] The Fischer Family Trust[11] (FFT) identified pupil records for the trial participants in 2018 on the basis of identifying information collected at randomisation and during periods of active follow-up (**Figure 2, Supplementary Table 2**).

Interventions

The seven parallel group trials investigated the superiority of different infant formula modifications over standard formula in different infant populations (**Figure 3**). The formula compositions for each trial are detailed in the supplementary material. All but two of the interventions (sn-2 Palmitate and Nucleotides) were assumed to have an effect on long-term cognitive function. All analyses will be stratified by trial as effect mechanisms might differ.

For peer review only - http://bmjopen.bmj.com/site/about/guidelines.xhtml

Outcomes

Primary outcome

At age 16 years (during Key Stage 4), English pupils take their General Certificate of Secondary Education (GCSE). The primary outcome for this study will be the mean difference in GCSE mathematics exam z-score between control and intervention groups. GCSE mathematics exams are compulsory, nationally administered and are graded from 58 points to 0 points (**Table 1**).

Table 1 GCSE Grade Structure during analysis period (2001-2016)

Grade	Points equivalent	Remarks
A*	58	Highest pass
A	52	
B	46	
C	40	
D	34	
E	28	
F	22	
G	16	Lowest pass
U	0	Ungraded

We chose Mathematics over English, which is also compulsory and nationally administered, because exam results for mathematics are commonly considered to be less subjectively graded.[12] We chose the primary endpoint at age 16 rather than age 11 as it is a more relevant predictor of future education and employment opportunities. Children without KS4 will not be included in the primary outcome.

Secondary outcomes

As secondary outcomes we will investigate intervention effects of modified infant formula vs control formula on:

For peer review only - http://bmjopen.bmj.com/site/about/guidelines.xhtml

- mean GCSE English language exam z-scores
- mean Mathematics and English reading exam z-scores at age 11 (Key Stage 2, final year of primary school).
- probability of receiving five or more GCSEs with grades A* to C which includes Mathematics and English. (This is a critical accountability measure for schools, and is used in school performance tables).
- probability of receiving special educational needs (SEN) support

All mentioned Mathematics and English exams are compulsory and nationally administered. GCSE English language scores are graded like GCSE mathematic scores. Mathematics exams at age 11 are graded from 0–100, with 100 being highest possible score. English reading exams at age 11 are graded from 0–50, with 50 being highest. Receiving five or more GCSEs with grades A* to C is a commonly reported measure of academic performance as this measure feeds into entry requirements for a large number of sixth form colleges and therefore determines future academic development. Because the effect of the intervention on exam results might be mediated by special needs status, we also aim to determine whether the intervention affects the probability of receiving SEN support.

All z-scores will be calculated as:

$$\text{z-score} = \frac{x - \mu}{SD_{pooled}}$$

Where SD_{pooled} is the pooled standard deviation of the respective exam point scores from control and intervention groups within each trial.

Probabilities will be reported as odds of having the outcome in the intervention group over odds of having the outcome in the control group within each trial.

Covariates and interactions

To increase the statistical efficiency of our analysis we will adjust for covariates that have a strong association with the analysis outcome and were measured at randomisation (i.e. are not on the causal pathway between intervention and outcome). On the basis of previous internal analyses using linked *Millennium Cohort Study* and NPD data as well as previously published studies[5 13 14] we expect maternal smoking and maternal education as well as birthweight and

For peer review only - http://bmjopen.bmj.com/site/about/guidelines.xhtml

gestational age to be strongly associated with educational performance. Analyses involving multi-centre trials with separate randomisation schedules for each centre will also be adjusted for centre. [15] However, not every covariate was measured in all trials (**Table 2**).

Table 2 Variables a-priori expected to be associated with educational performance

Trial	Centre	Gestational age	Birthweight	Maternal smoking	Maternal education
Type	*Nominal*	*Continuous*	*Continuous*	*Binary*	*Ordinal*
RCT 1 1993/96 Nutrient-enriched post-dc.	X	X	X	X	X
RCT 2 1993/96 Nutrient-enriched (SGA)	X	X	X	X	X
RCT 3 1993/96 LCPUFA (preterm)	X	X	X	X	X
RCT 4 1993/95 LCPUFA (term)	X	X	X	X	X
RCT 5 2000/02 Nucleotides	X	X	X	X	X
RCT 6 1993/94 Iron	X	X	X	X	X
RCT 7 1995/96 Sn-2 palmitate	n/a	X	X	X	X

n/a not applicable; *LCPUFA* Long chain polyunsaturated fatty acids, *SGA* born small for gestational age, *post-dc.* formula after discharge from hospital

Based on reports from previous studies[16] we also hypothesise that the effect of supplementations will depend on sex and whether the mother smoked during pregnancy or not. However, the trials were not powered to measure this interaction; therefore, we will

For peer review only - http://bmjopen.bmj.com/site/about/guidelines.xhtml
For peer review only

include an interaction term in an exploratory analysis so the results can be included in future meta-analyses.

Statistical methods

All statistical analyses will be conducted using Stata version 16.[17]

Primary analysis

For our primary analysis we will use multivariable regression models to compare intervention and control groups stratified by trial and adjusted for a-priori determined risk factors (**Table 2**). We will present effect estimates as mean differences with 95% confidence intervals. The analysis will be on intention-to-treat basis. The primary analysis can be expressed using the following equation:

$$\text{Z-}score_{GCSE\ Maths} = \beta_0 + \beta_1 \times centre + \beta_2 \times sex + \beta_3 \times birthweight + \ldots + \beta_p \times control\ milk + e$$

Where β_0 (intercept) is the z-score of GCSE Mathematics when all predictors are zero and e refers to the residual terms. This model assumes that GCSE Mathematics scores are linearly related to the other predictors. If for a given predictor the relationship it is not linear, we will account for this by categorising quantitative variables or including quadratic terms. We will also check that there is no multicollinearity between the predictors, residuals are residuals are consistent with random error and independent and that the variance is constant across the outcome and the predictors.

Secondary analyses

Mean GCSE English language exam z-scores, and mean Mathematics and English reading exam z-scores at age 11 will be analysed in the same way as the primary outcome.
The probability of receiving five or more GCSEs with grades A* to C and the probability of receiving SEN support will be modelled use logistic regression, and adjusted for the same factors as the primary outcome (**Table 2**). We will take into account the risk of false positives arising from multiple testing when interpreting the results. Line graphs will be used to visually explore Mathematics and English exam z-scores and their 95% confidence intervals at age 16 and 11, by trial and trial arm.

For peer review only - http://bmjopen.bmj.com/site/about/guidelines.xhtml

Sensitivity analyses

To determine the effect of the intervention on GCSE Mathematics scores in context of the general population, we will calculate the z-scores using national standard deviations rather than the trial specific ones. Due to the higher heterogeneity in the national sample, we expect our effect estimate to be more conservative in this analysis.

In order to assess the robustness of our analysis, we will also present the unadjusted outcomes as a sensitivity analysis, and model the outcome using an ordered logit model adjusting for the above mentioned covariates (**Table 2**).

To assess the sensitivity of our end-point measure, we are going to present results for the cohort of non-randomised breast-fed recruits from the trials alongside the randomised groups. Breastfed children have been consistently reported to have better cognitive ability and perform better in school than their formula-fed peers.[18 19] Therefore, we also expect to observe better school performance for previously breast-fed compared to formula-fed children in the primary outcome. Breastfed children were recruited in all trials except for the 1993/94 Iron trial. Additionally, we expect there to be no effect of sn-2 palmitate enriched formula and included this trial cohort to further validate our approach.

Exploratory analyses

While this study is underpowered to determine interaction effects, we intend to conduct exploratory analyses stratified by sex and smoking status during pregnancy, so these are available for inclusion in future meta-analyses.

Power

The primary outcomes (mean difference in z-score of mathematics exam at age 16) will be continuous and analyses will be conducted for each trial separately. We expect primary outcomes to be available for around 70% of the original cohort with an equal retention rate in treatment and control arms. A 0.1 standard deviation (SD) difference[*] will be regarded as clinically significant.[20 21] The desired power to detect a difference between the treatment arms

[*] this corresponds to 1/5th of a GCSE grade or 1.154 points on the 58 points point scale.

For peer review only - http://bmjopen.bmj.com/site/about/guidelines.xhtml

that is at least as large as this, is 80% with a significance level of 0.05. The R-squared is expected to be 0.05-0.1. Taking the 1993/95 LCPUFA term study (n=309) as an example, a conservative 50% (n=154) linkage success would enable a detection of 0.45 SD difference (e.g. 9/10 of a grade or 5.193 grade points).

Missing data

For our primary analysis we will use multiple imputation to impute missing covariates using 15 imputations and setting the random seed at 300.[15] We will use Stata's *mi chained* command to perform the imputation. We will also perform sensitivity analyses to determine the potential impact of missing outcome data by looking at worst case scenarios (i.e. all missing participants with missing outcome data achieved the lowest grade/ highest grade possible).

Assessment of bias

For each trial, we will compile information for a Cochrane risk of bias analysis.[22]
To detect whether selection bias at follow-up has affected the randomisation balance of characteristics we will present the pre-randomisation characteristics as percentage/ mean differences between intervention and control groups at randomisation and at age 16 (when the primary outcome is measured) with 95% confidence intervals.

Discussion

Expected outcomes of the study

High participant retention rates are essential to determine delayed intervention effects that might not become apparent in short-term follow-ups or when using traditional follow-up methods which typically suffer from high attrition.[3 23] Extending follow-up of RCTs without the need for participants to re-engage is possible where trial data and participant identifiers have been retained and governance arrangements allow secure linkage that safeguards participants' privacy. Post-trial extension using opt-out linkage to administrative data will be especially desirable for future research if it reduces costs associated with conventional methods of follow-up (such as face-to face interviews) and simultaneously improves participant retention rates (compared to opt-in data linkage). Critically, our study will allow new insights into the long-term cognitive benefits and safety profile of modified infant

For peer review only - http://bmjopen.bmj.com/site/about/guidelines.xhtml

formulas, by making use of randomised historical infant formula comparisons which, are unlikely to be investigated in future RCTs either due to lack of equipoise or resources.

Limitations

Our study should be considered in the context of several limitations.

First, only schools following the national curriculum have to contribute data to the NPD, while independent schools may provide data voluntarily. [24] Some participants who received their education outside state-funded English schools will therefore be excluded from this analysis. In any given academic year, however, data from 99% of all children of compulsory school age are included in the NPD database [10]. Consequently, we expect this to have minimal, non-differential impact on overall linkage success.

Second, there is no general agreement on what defines educational performance and which age is optimal to capture intervention effects. To minimise missing data and detect potential trajectory effects, we will therefore rely on data from two high-stakes examinations sat by the majority of state school students in England.

Third, linkage quality plays a role when using administrative data to follow up participants.[25-28] The FFT will provide us with up to three different pupil records per participants (based on names, date of birth and location). We will use probabilistic methods adapted from Fellegi and Sunter[29] as well as manual review to decide which record, if any, we consider to be the best match (**Supplementary Material**).

Fourth, we acknowledge that intervention effects measured 15 years later might be diluted by contextual factors. Convergence of trial arms may result from catch-up over time as those who lag behind in early years might receive extra (e.g. educational) assistance. There will also be variability in terms of quality of education. However, this is likely to be roughly equal between intervention and control groups due to adequate sample size, randomisation, and blinding. Furthermore, theories of early programming suggest that early postnatal life is a critical period of development[30] and nutritional stimuli during this period can result in lifelong permanent effects[31]. If true, any effective nutritional intervention is unlikely to disappear completely by early adulthood. If not, our study should be of increased interest to the scientific community concerned with the early origins of adult health and disease theory.

For peer review only - http://bmjopen.bmj.com/site/about/guidelines.xhtml

Either way, it will provide important insights for parents, policy makers, and the scientific community as to whether choice of infant formula type can have long-lasting, measurable effects on something as important as educational performance.

Ethics and dissemination

Ethics approval and legal basis for data processing

The proposal to link the trials and NHS data underwent a robust ethical, legal, and technical review. The proposed project was approved by the UCL Research & Development Office and Data Protection Office (Reference: 14PE15), the NHS London-City & East Research Ethics Committee (Reference: 17/LO/0556) as well as the Health Research Authority and Health and Care Research Wales (Reference: 212148). An exemption under Section 251 of the NHS Act 2006 was granted by the Confidentiality Advisory Group (Reference: 17CAG0051) and allows UCL GOS ICH and the FFT to process identifiable participant information to facilitate the data linkage and analyses without consent. Dissent and fair processing were discussed with the HRA CAG and an opt-out procedure is in place.[32]

Confidentiality

Special efforts are made protect participant privacy and confidentiality. All linked data will be de-identified and thus contain no information on personal identifiers such as names and addresses. Identifiers will be stored at separate access restricted locations certified to international standards (ISO/IEC 27001). Access to all data will also be physically restricted. Analysis data will be stored as aggregated as possible (such as school types instead of school IDs), to minimise the risk of re-identification. All analyses will be conducted in a digital safe haven and no individual level data is allowed to leave the server. Outputs will have to meet statistical disclosure controls that prevent small cell sizes to be exported from the server. Additionally, access to the data will be restricted to an authorised group of researchers, who will all have undergone the HSCIC and ADRN information governance training and are bound by their university contracts to treat the data confidentially.

Project management

The Fischer Family Trust links the data which is then securely transferred to the UCL data safe haven. MV will clean and analyse the linked data and draft the initial manuscript. KH and RG will advise on data cleaning and contribute to the interpretation of results. JJ will

For peer review only - http://bmjopen.bmj.com/site/about/guidelines.xhtml

contribute to interpreting exam results and MF will provide background information on the trials and contribute to interpreting potential effects of the modified formula. All authors will contribute to writing the final manuscript.

Dissemination of results and publication policy

To enhance the use, understanding, and dissemination of this study, the results will be disseminated to both scientific and lay audiences, submitted for publication in peer-reviewed journals, and will contribute towards a PhD dissertation.

Funding

MV is supported by the Economic and Social Research Council (ESRC) and the Great Ormond Street Hospital Charity. RG and KH receive funding from Health Data Research UK, an initiative funded by the UK Research and Innovation, Department of Health and Social Care (England) and the devolved administrations, and leading medical research charities. KH receives funding from Wellcome (grant 103975/Z/14/Z). The funding sources had no role in study design, write-up or decision to publish.

Footnotes

Author Statement:

MV has prepared the initial draft of the protocol. KH, JJ, MF and RG provided substantial intellectual input. All authors have contributed to writing the final draft of the manuscript.

Competing interests:

Mary Fewtrell has been a member of the Infant Nutrition Working Group at EFSA (European Food Safety Authority) since 2013. She was involved in data analysis and publication of randomised trials of LCPUFA-supplemented infant formulas funded by grants from Numico Res BV and Heinz UK. The companies also provided the infant formulas for the studies. She was also involved in follow-up studies (including cognitive outcome) of children and adolescents from randomised trials of LCPUFA-supplemented formulas, with funding from the Medical Research Council and European Union (FP6-FOOD-2005-007036). Maximiliane Verfuerden, Katie Harron, John Jerrim, and Ruth Gilbert have no competing interests to declare.

For peer review only - http://bmjopen.bmj.com/site/about/guidelines.xhtml

Data sharing statement:

Statistical code will be available from GitHub. Participant-level data will be analysed under licence and are not available for sharing. Aggregate, non-identifiable data are available upon reasonable request.

Ethics and patient consent:

This study has been approved by NHS Confidentiality Advisory Group (17CAG0051) and the London - City & East Research Ethics Committee (17/LO/0556). Patient consent for publication was not required.

Patient and public involvement:

We consulted with a subset of former trial participants about the acceptability of our study design and received strong support for our approach. We used the discussions to improve our reporting and dissemination strategy.

For peer review only

For peer review only - http://bmjopen.bmj.com/site/about/guidelines.xhtml

References

1. Sun H, Como PG, Downey LC, et al. Infant formula and neurocognitive outcomes: impact of study end-point selection. *Journal Of Perinatology* 2015;35:867. doi: 10.1038/jp.2015.87

https://www.nature.com/articles/jp201587#supplementary-information

2. Sun H, Como PG, Downey LC, et al. Infant formula and neurocognitive outcomes: Impact of study end-point selection. *Journal of Perinatology* 2015;35(10):867-74.

3. Fewtrell MS, Domellof M, Hojsak I, et al. Attrition in Long-Term Nutrition Research Studies: A Commentary by the European Society for Pediatric Gastroenterology, Hepatology, and Nutrition Early Nutrition Research Working Group. *Journal of pediatric gastroenterology and nutrition* 2016;62(1):180-2. doi: 10.1097/mpg.0000000000000992 [published Online First: 2015/09/30]

4. Chmielewska A, Dziechciarz P, Gieruszczak-Białek D, et al. Effects of prenatal and/or postnatal supplementation with iron, PUFA or folic acid on neurodevelopment: update. *Br J Nutr* 2019;122(s1):S10-s15. doi: 10.1017/s0007114514004243 [published Online First: 2019/10/23]

5. Sammons P, Sylva K, Melhuish E, et al. Influences on students' GCSE attainment and progress at age 16. *Department for Education* 2014

6. Deary IJ, Strand S, Smith P, et al. Intelligence and educational achievement. *Intelligence* 2007;35(1):13-21.

7. Borghans L, Golsteyn BHH, Heckman JJ, et al. What grades and achievement tests measure. *Proceedings of the National Academy of Sciences of the United States of America* 2016;113(47):13354-59. doi: 10.1073/pnas.1601135113 [published Online First: 2016/11/08]

8. Llewellyn-Bennett R, Edwards D, Roberts N, et al. Post-trial follow-up methodology in large randomised controlled trials: a systematic review. *Trials* 2018;19(1):298. doi: 10.1186/s13063-018-2653-0 [published Online First: 2018/05/31]

9. Boyd A, Golding J, Macleod J, et al. Cohort Profile: the 'children of the 90s'--the index offspring of the Avon Longitudinal Study of Parents and Children. *International journal of epidemiology* 2013;42(1):111-27. doi: 10.1093/ije/dys064 [published Online First: 2012/04/18]

10. Jay MA, Mc Grath-Lone L, Gilbert R. Data Resource: the National Pupil Database (NPD). *International Journal of Population Data Science* 2019;4(1)

11. FFT Education Ltd. Fischer Family Trust 2019 [Available from: https://fft.org.uk/ accessed July 2019.

12. Rhead S, Black B. Marking consistency metrics: An update. In: Ofqual, ed. Coventry: Ofqual, 2018.

For peer review only - http://bmjopen.bmj.com/site/about/guidelines.xhtml

13. Botting N, Powls A, Cooke RW, et al. Cognitive and educational outcome of very-low-birthweight children in early adolescence. *Developmental Medicine & Child Neurology* 1998;40(10):652-60. doi: 10.1111/j.1469-8749.1998.tb12324.x

14. Abel K, Heuvelman H, Wicks S, et al. Gestational age at birth and academic performance: population-based cohort study. *International journal of epidemiology* 2017;46(1):324-35. doi: 10.1093/ije/dyw284 [published Online First: 2016/11/08]

15. European Medicines Agency. Guideline on Missing Data in Confirmatory Clinical Trials 2010.

16. de Jong C, Kikkert HK, Fidler V, et al. Effects of long-chain polyunsaturated fatty acid supplementation of infant formula on cognition and behaviour at 9 years of age. *Developmental medicine and child neurology* 2012;54(12):1102-8. doi: 10.1111/j.1469-8749.2012.04444.x [published Online First: 2012/10/17]

17. Stata Statistical Software: Release 16. [program]. College Station, TX: StataCorp LLC, 2019.

18. Victora CG, Horta BL, de Mola CL, et al. Association between breastfeeding and intelligence, educational attainment, and income at 30 years of age: a prospective birth cohort study from Brazil. *The Lancet Global Health* 2015;3(4):e199-e205. doi: 10.1016/S2214-109X(15)70002-1

19. Lucas A, Morley R, Cole TJ, et al. Breast milk and subsequent intelligence quotient in children born preterm. *Lancet (London, England)* 1992;339(8788):261-4. doi: 10.1016/0140-6736(92)91329-7 [published Online First: 1992/02/01]

20. Fryer Jr RG. The production of human capital in developed countries: Evidence from 196 randomized field experiments. Handbook of economic field experiments: Elsevier 2017:95-322.

21. Kraft MA. Interpreting Effect Sizes of Education Interventions. *Educational Researcher*

22. Higgins JP, Altman DG, Gøtzsche PC, et al. The Cochrane Collaboration's tool for assessing risk of bias in randomised trials. *Bmj* 2011;343:d5928.

23. Fewtrell MS, Kennedy K, Singhal A, et al. How much loss to follow-up is acceptable in long-term randomised trials and prospective studies? *Arch Dis Child* 2008;93(6):458-61. doi: 10.1136/adc.2007.127316 [published Online First: 2008/05/23]

24. Department for Education. Freedom of Information Request by Jen Persson 2017 [Available from: https://www.whatdotheyknow.com/request/pupil_data_national_pupil_databa_3?unfold=1 accessed 28 Mar 2020.

25. Harron K, Dibben C, Boyd J, et al. Challenges in administrative data linkage for research. *Big Data & Society* 2017;4(2):2053951717745678. doi: 10.1177/2053951717745678

26. Harron K, Wade A, Gilbert R, et al. Evaluating bias due to data linkage error in electronic healthcare records. *BMC Medical Research Methodology* 2014;14(1):36. doi: 10.1186/1471-2288-14-36

For peer review only - http://bmjopen.bmj.com/site/about/guidelines.xhtml

27. Harron KL, Doidge JC, Knight HE, et al. A guide to evaluating linkage quality for the analysis of linked data. *International journal of epidemiology* 2017;46(5):1699-710. doi: 10.1093/ije/dyx177 [published Online First: 2017/10/13]

28. Harron K, Wade A, Muller-Pebody B, et al. Opening the black box of record linkage. *J Epidemiol Community Health* 2012;66(12):1198. doi: 10.1136/jech-2012-201376 [published Online First: 2012/06/19]

29. Fellegi IP, Sunter AB. A Theory for Record Linkage. *Journal of the American Statistical Association* 1969;64(328):1183-210. doi: 10.1080/01621459.1969.10501049

30. Barker DJ, Osmond C. Infant mortality, childhood nutrition, and ischaemic heart disease in England and Wales. *The Lancet* 1986;327(8489):1077-81.

31. Lucas A. Long-term programming effects of early nutrition -- implications for the preterm infant. *J Perinatol* 2005;25 Suppl 2:S2-6. doi: 10.1038/sj.jp.7211308 [published Online First: 2005/04/30]

32. UCL Great Ormond Street Institute of Child Health. Information for participants 2020 [Available from: https://www.ucl.ac.uk/child-health/research/population-policy-and-practice/research/studies/head-or-heart-study.

Figure legends/ captions:

Figure 1: Combined flow of participants of seven infant formula trials

Figure 2: Data flows

(1) Participant identifier information securely transferred for linkage to FFT. (2) Deterministic linkage at FFT, application of agreement flags to all candidate pairs, and candidate pairs securely sent to UCL minus participant identifiers. (3) Implausible pairs discarded using RCT clinical variables (linked through unique study ID number). (4) Probabilistic linkage at UCL. (5) Data cleaning. UCL University College London; RCT randomised controlled trial; NPD National pupil data base, FFT Fischer-Family Trust.

Figure 3: Trial interventions, comparators and populations

For peer review only - http://bmjopen.bmj.com/site/about/guidelines.xhtml

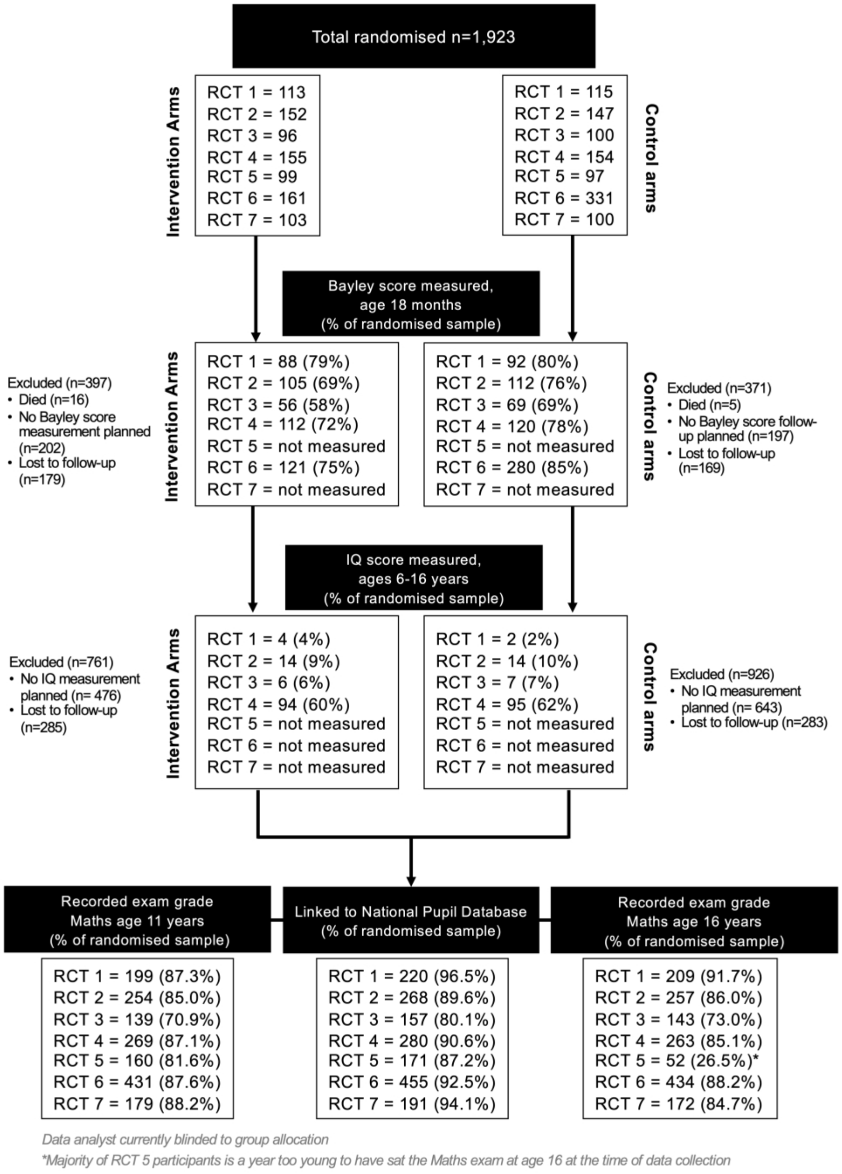

Figure 1 Combined flow of participants of seven infant formula trials

126x175mm (800 x 800 DPI)

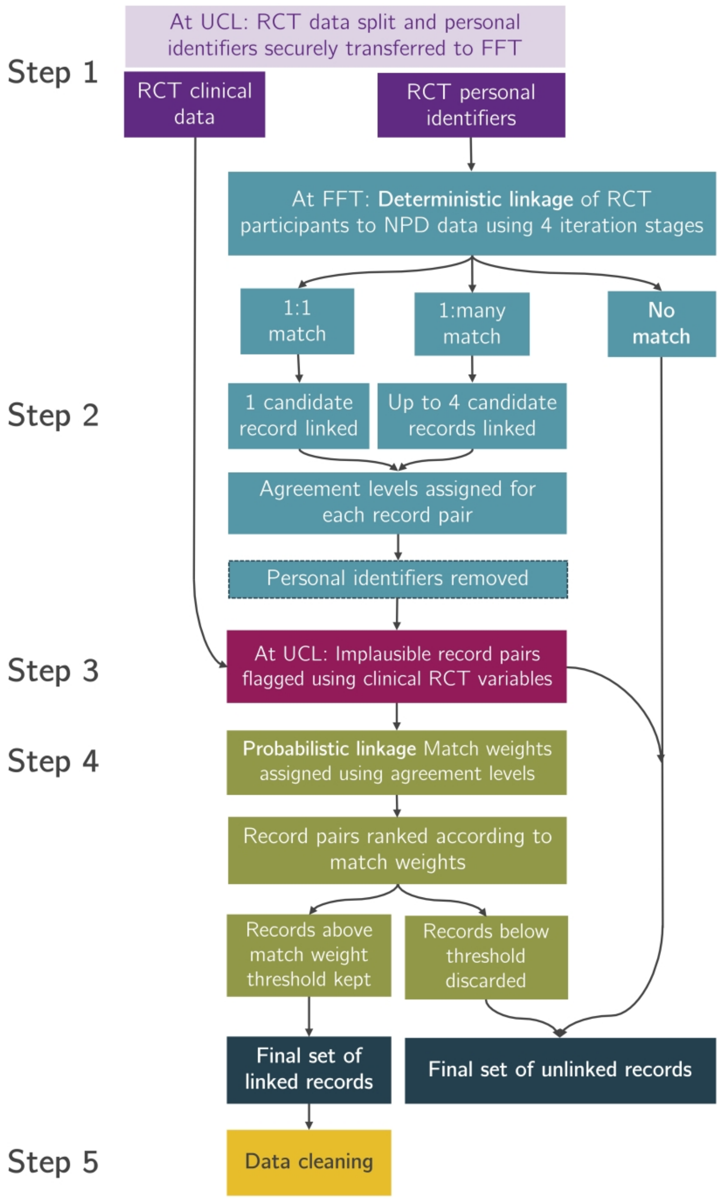

Figure 2 Data Flows. (1) Participant identifier information securely transferred for linkage to FFT. (2) Deterministic linkage at FFT, application of agreement flags to all candidate pairs, and candidate pairs securely sent to UCL minus participant identifiers. (3) Implausible pairs discarded using RCT clinical variables (linked through unique study ID number). (4) Probabilistic linkage at UCL. (5) Data cleaning. UCL University College London; RCT randomised controlled trial; NPD National pupil data base, FFT Fischer-Family Trust.

127x208mm (900 x 900 DPI)

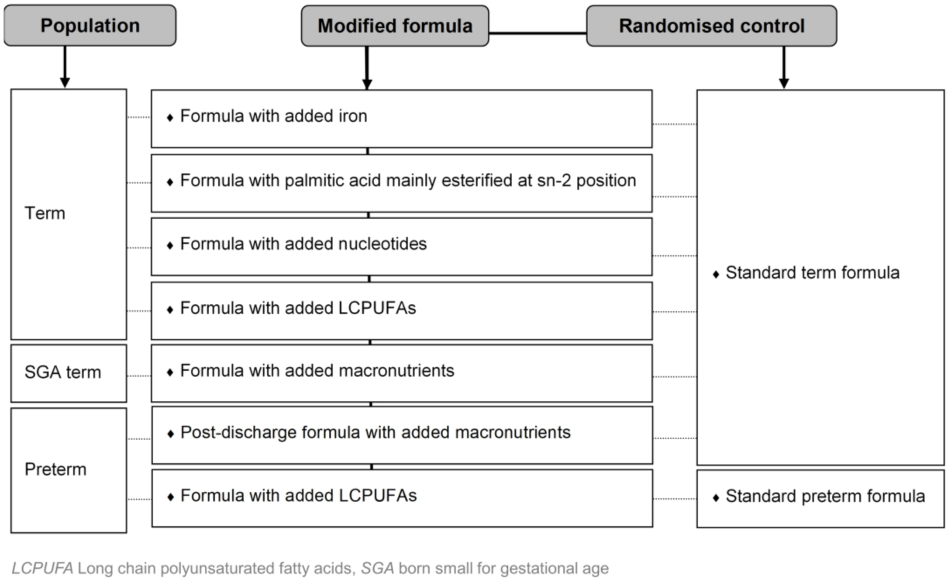

Figure 3 Trial interventions, comparators and population

351x230mm (300 x 300 DPI)

bmjopen-2019-035968 R.2 Supplementary material

Supplementary Table 1 Trial Characteristics

	RCT 1	RCT 2	RCT 3	RCT 4	RCT 5	RCT 6	RCT 7
Intervention	Nutrient-enriched	Nutrient-enriched	LCPUFA	LCPUFA	Nucleotides	Iron	Sn-2 palmitate
Population	Preterm	SGA Term	Preterm	Term	Term	Term	Term
Years randomised	1993-96	1993-96	1993-96	1993-95	2000-02	1993-94	1995-96
Eligibility	<1750g bw <37w ga	bw <10th centile ≥37w ga	≤1750g bw ≤37w ga	≥37w ga	≥37w ga	bw > 2500g, ≥37w ga age: 9m	≥37w ga, bw>5th centile
Control formula:	Term formula	Term formula	Preterm formula	Term formula	Term formula	Term formula or cow's milk	Term formula
Intervention: Control formula with added:	Macro nutrients and vitamins	Macro nutrients and vitamins	DHA and AA	DHA and AA	Nucleotides	12 mg/L iron	Sn-2 palmitate
Timing of intervention	1w < discharge - 9m	Birth – 12w	Birth – 3w	Birth – 6m	Birth – 20w	9m - 18m	Birth – 12w
Centre(s)	Nottingham Leicester Cambridge Ipswich	Nottingham Leicester Cambridge	Nottingham Leicester	Nottingham Leicester	Nottingham Leicester	Nottingham Leicester Norwich	Cambridge
Total N	296	475	284	447	296	492	323
Intervention	113	152	96	155	99	161	103
Control (1)	115	147	100	154	97	165	100
Control (2)	-	-	-	-	-	166	-
Non-randomised breastfed reference	68	176	88	138	100	-	120
*% Response at last cognitive follow-up (test, age) **	84% (Bayley score, 18m)	6% (IQ score, 17 years)	9% (IQ score, 16 years)	55% (IQ score, 5.5 years)	81% (Head circumference, 20 weeks)	36% (Bayley score, 18m)	No cognitive follow-up
References	1	2 3	4 5	6 7	8 9	10 11	12 13

bw=birthweight, ga=gestational age, d=days, w=week(s), m=moths, LCPUFA=Long Chain Polyunsaturated Fatty Acids, SGA=small-for-gestational-age, m=months, DHA= docosahexaenoic acid, AA= arachidonic acid *includes unpublished follow-ups

For peer review only

bmjopen-2019-035968 R.2 Supplementary material

Data cleaning methods:

The party who conducts the linkage (the Fischer Family Trust, FFT) provides up to three likely matching pupil IDs and their school records per participant. Only one of these records can be a true match and it is possible that none of the linked pupil IDs is a true match, as participants might have emigrated, died or attended private schools which do not transmit data to the national pupil database. We will check and cross validate the data provided by FFT extensively, using auxiliary variables from the trials (such as known death) and linkage confidence flags supplied by FFT (Supplementary Table 2).

Supplementary Table 2 Possible match levels

Identifier	Description
First name	First name and other first name both exact match
	First name matches other name in both directions
	First name exact match
	First name matches other name
	Other name exact match
	First name truncated at any hyphen matches
	First name matches via common name alternatives lookup
	Pattern match function
	Pattern match function - AND first character of first name matches
	First name / surname match in both directions
	No Link
Surname	Surname exact match (including alternative surnames)
	Surname truncated at any hyphen matches
	Pattern match function
	Pattern match function - AND first character of surname matches
	First name / surname match in both directions
	No Link
Date of Birth	DOB exact match
	Day on source matches month on match, and vice versa; year matches (i.e. transposed date)
	Day and month match (i.e. wrong year)
	Day and year match (i.e. wrong month)
	Month and year match (i.e. wrong day)
	Either source or match DOB is 1st January; year matches
	Either source or match DOB is 1st September; year matches
	No Link
Location	Postcode exact match
	Local Authority match
	Neighbouring / nearby Local Authority match

For peer review only - http://bmjopen.bmj.com/site/about/guidelines.xhtml

bmjopen-2019-035968 R.2 Supplementary material

No Link

We will calculate match-weights for each participant-pupil pair according to a probabilistic method adapted from Fellegi and Sunter.[14] These match-weights will then be used to rank the pairs according to their probability of being a true match. For each match category (Supplementary Table 2) we will derive proxy-m and proxy-u probabilities which will then be used to produce the match weight.

M-probability: probability of agreeing on a category given that it is a true match
U-probability: probability of agreeing on a category given by chance

The original method requires a set of participant-pupil pairs that are certain true matches and a set of participant-pupil pairs that are certain false matches. Due to the de-identified nature of the data, such information will not be available and thus needs to be approximated. True matches will be defined as participants perfectly matching all categories (except the one we are calculating the probability for). Certain false matches will be defined as participants disagreeing in all categories (except the one we are calculating the probability for). This will yield a total of eight reference datasets. For each linkage flag (sex, first name, last name, date of birth, location) we will then calculate the variable weight using:

$$\log 2\ (m/u)$$

The final match weight for a participant-pupil pair will be calculated using the sum of all weights in a pair:

$$\sum \log 2\ (m/u)$$

At this stage each participant will still have up to three multiple possible matches.
First, we will drop all participants that linked but where their age at first link was >17 and they do not agree with the record on date of birth. This is because the date of birth in the trial data is very likely to be correct as it corresponds to the recruitment periods and was confirmed repeatedly during the follow-up period. We will also discard all records for children that died before school age (4 years) where information on death and time of death during the trial period is available.

We will then rank participant-pupil pairs according to their match weights. If the highest match-weight for a participant is 10% above that of the second best match, we will automatically keep the best match and discard the other(s). If the difference is lower, we will manually review these

For peer review only - http://bmjopen.bmj.com/site/about/guidelines.xhtml

records to decide which one is the best match (if any). Decisions taken at manual review will be reported to ensure reproducibility. We will then choose two cut-offs based on visual inspection of the match probabilities; one threshold to maximise sensitivity (many true positive links) and one to maximise specificity (few false positive links). To determine how representative each sample defined by these cut-offs is of the original randomised cohort, we will compare them across the variables that were measured at randomisation. We will use the bigger, more sensitive sample in our analyses but use the smaller, more specific sample in sensitivity analyses.

For peer review only

bmjopen-2019-035968 R.2 Supplementary material

Trial formula compositions:

RCT 1: Preterm nutrient enriched formula (post-discharge) vs standard formula

per 100 ml	Standard formula	High-energy formula
Energy (kcal)	68	72
Protein (g)	1.45	1.85
Casein	0.56	0.72
Whey	0.89	1.13
Carbohydrate (g)	6.96	7.24
Lactose (g)	6.96	6.20
Maltodextrin (g)	-	1.04
Fat (g)	3.82	3.96
Minerals		
Calcium (mg)	39	70
Phosphorus (mg)	27	35
Sodium (mg)	17	22
Chloride (mg)	45	45
Potassium (mg)	57	78
Iron (mg)	0.65	0.65
Zinc (mg)	0.34	0.60
Copper (µg)	42	57
Iodine (µg)	4.5	4.5
Magnesium (mg)	5.2	5.2
Manganese (µg)	3.4	5
Vitamins		
Vitamin A (µg)	100	100
Thiamine B_1 (mg)	42	95
Riboflavin B_2 (mg)	55	100
Niacin B_3 (µg)		
Pantothenic acid B_5 (mg)	0.23	0.40
Pyridoxine B_6 (µg)	35	80
Biotin B_7 (µg)	1.0	1.1
Inositol B_8 (mg)		
Folate B_9 (µg)	3.4	25
Cyanocobalamin B_{12} (µg)	0.14	0.2
Vitamin C (mg)	6.9	15
Vitamin D (µg)	1.0	1.3
Vitamin E (mg)	0.48	1.5
Vitamin K (µg)	2.7	6.0
Choline (mg)	4.8	5.1
Taurine (mg)	5	5.1
Carnitine (mg)	-	1.1

For peer review only

bmjopen-2019-035968 R.2 Supplementary material

RCT 2: Term SGA nutrient enriched formula vs standard formula

per 100 ml	Standard formula	High-energy formula
Energy (kcal)	68	72
Protein (g)	1.45	1.85
Casein	0.56	0.72
Whey	0.89	1.13
Carbohydrate (g)	6.96	7.24
Lactose (g)	6.96	6.20
Maltodextrin (g)	-	1.04
Fat (g)	3.82	3.96
Minerals		
Calcium (mg)	39	70
Phosphorus (mg)	27	35
Sodium (mg)	17	22
Chloride (mg)	45	45
Potassium (mg)	57	78
Iron (mg)	0.65	0.65
Zinc (mg)	0.34	0.60
Copper (µg)	42	57
Iodine (µg)	4.5	4.5
Magnesium (mg)	5.2	5.2
Manganese (µg)	3.4	5
Vitamins		
Vitamin A (µg)	100	100
Thiamine B_1 (mg)	42	95
Riboflavin B_2 (mg)	55	100
Niacin B_3 (µg)		
Pantothenic acid B_5 (mg)	0.23	0.40
Pyridoxine B_6 (µg)	35	80
Biotin B_7 (µg)	1.0	1.1
Inositol B_8 (mg)		
Folate B_9 (µg)	3.4	25
Cyanocobalamin B_{12} (µg)	0.14	0.2
Vitamin C (mg)	6.9	15
Vitamin D (µg)	1.0	1.3
Vitamin E (mg)	0.48	1.5
Vitamin K (µg)	2.7	6.0
Choline (mg)	4.8	5.1
Taurine (mg)	5.0	5.1
Carnitine (mg)	-	1.1

RCT 3: Preterm LCPUFA-enriched formula vs standard preterm formula

per 100 ml	Standard formula	LCPUFA-enriched formula
Energy (kcal)	70	70

For peer review only

Protein (g)	2.0	2.0
Casein	0.8	0.8
Whey	1.2	1.2
Carbohydrate (g)	7.7	7.7
Total fat (g)	3.5	3.5
Fatty acid composition (g/ 100 g fat)		
C8:0 caprylic	0.7	0.6
C10:0 capric	1.2	1.1
C12:0 lauric	6.3	4.9
C14:0 myristic	5.6	5.6
C16:0 palmitic	25.8	26.3
C18:0 stearic	8.2	8.5
C18:1 oleic	32.6	32.9
C18:2 n-6 linoleic	10.6	12.0
C18:3 n-6 γ-linolenic	0.1	0.4
C18:3 n-6 α-linolenic	0.7	0.6
C20:0 arachidic	0.4	0.3
C20:1 n-9 eicosanoic	0.2	0.3
C20:4 n-6 AA	-	0.31
C20:5 n-3 eicosapentaenoic	-	0.04
C22:0	0.3	0.2
C22:6 n-3 DHA	-	0.17
C24:0	0.2	0.1
Other fatty acids	7.1	4.62
Cholesterol	-	7.73
Minerals		
Calcium (mg)	70	70
Phosphorus (mg)	35	42
Sodium (mg)	30	27
Potassium (mg)	75	71
Iron (mg)	0.1	0.07
Zinc (mg)	0.39	0.4
Vitamins		
Vitamin A (µg)	63	63
Vitamin D (µg)	2.1	2.1
Vitamin E (mg)	2.0	2.0
Vitamin K (µg)	2.8	3.0

RCT 4: Term LCPUFA-enriched formula vs standard term formula

per 100 ml	Standard formula	LCPUFA-enriched formula
Energy (kcal)	67	67
Protein (g) (casein:whey 40:60)	1.5	1.5
Carbohydrate (g)	7.6	7.7
Lactose (g)	7.6	7.7
Total fat (g)	3.4	3.4
Fatty acid composition (% total fat)		
C8:0 caprylic	0.8	2.0
C10:0 capric	2.4	2.0

For peer review only

C12:0 lauric	1.6	12.3
C14:0 myristic	8.6	5.2
C16:0 palmitic	23.3	25.6
C18:0 stearic	10.2	4.6
C18:1 oleic	32.7	29.7
C18:2 n-6 linoleic	12.4	15.9
C18:3 n-6 α-linolenic	1.1	1.4
C20:4 n-6 AA	-	0.30
C20:5 n-3 eicosapentaenoic	-	0.01
C22:6 n-3 DHA	-	0.32
Cholesterol	<0.5	0.8
Minerals		
Iron (mg)	0.8	0.8

RCT 5: Nucleotide-enriched formula vs standard formula

per 100 ml	Standard formula	Nucleotide-enriched formula
Energy (kcal)	68	68
Protein (g) (casein:whey 40:60)	1.5	1.5
Casein	0.6	0.6
Whey	0.9	0.9
Carbohydrate as lactose (g)	7.0	7.0
Total fat (g)	3.8	3.8
C18:2 n-6 linoleic (mg)	350	350
C18:3 n-6 γ-linolenic (mg)	33	33
C18:3 n-6 α-linolenic (mg)	44	44
LCPUFAs (mg)	26	26
Nucleotides		
Cytidine monophosphate (mg)	0.3	1.5
Uridine monophosphate (mg)	-	0.5
Adenosine monophosphate (mg)	-	0.6
Guanosine monophosphate (mg)	-	0.2
Inosine monophosphate (mg)	-	0.3
Minerals		
Calcium (mg)	39	39
Magnesium (mg)	5.2	5.2
Manganese (µg)	3.4	3.4
Phosphorus (mg)	27	27
Sodium (mg)	17	17
Potassium (mg)	0.3	0.3
Iron (mg)	0.6	0.6
Zinc (mg)	0.3	0.3
Copper (µg)	42	42
Iodine (µg)	4.5	4.5
Vitamins		
Vitamin A (µg)	100	100
Thiamine B_1 (mg)	42	42
Riboflavin B_2 (mg)	55	55

For peer review only - http://bmjopen.bmj.com/site/about/guidelines.xhtml

Niacin B_3 (mg)	0.7	0.7
Pantothenic acid B_5 (µg)	0.2	0.2
Biotin B_7 (µg)	1.0	1.0
Vitamin B_8 (µg)	35	35
Folic Acid B_9 (µg)	3.4	3.4
Cyanocobalamin B_{12} (µg)	0.14	0.14
Vitamin C (mg)	6.9	6.9
Vitamin D (µg)	1.0	1.0
Vitamin E (mg)	0.5	0.5
Vitamin K (µg)	2.7	2.7
Choline (mg)	4.8	4.8
Taurine (mg)	5.0	5.0

RCT 6: Iron-enriched formula vs standard formula

per 100 ml	Standard formula	Iron-enriched formula
Energy (kcal)	65	65
Protein (g)	2.5	2.5
Carbohydrate (g)	8.0	8.0
Lactose (g)	6.4	6.4
Maltodextrin	1.6	1.6
Fat (g)	2.8	2.8
Saturated (%)	42.9	42.9
Unsaturated (%)	57.1	57.1
Minerals		
Calcium (mg)	100	100
Phosphorus (mg)	65	65
Magnesium (mg)	7.5	7.5
Sodium (mg)	30	30
Potassium (mg)	100	100
Chloride (mg)	70	70
Iron (mg)	0.9	1.2
Zinc (mg)	0.5	0.5
Iodine (µg)	6.9	6.9
Vitamins		
Vitamin A (µg)	6.9	6.9
Thiamine B_1 (mg)	0.8	0.8
Riboflavin B_2 (mg)	0.12	0.12
Niacin B_3 (µg)	0.61	0.61
Pantothenic acid B_5 (µg)	0.24	0.24
Pyridoxine B_6 (µg)	48	48
Biotin B_7 (µg)	1.71	1.71
Inositol B_8 (mg)		
Folate B_9 (µg)	6.0	6.0
Cyanocobalamin B_{12} (µg)	0.12	0.12

For peer review only

bmjopen-2019-035968 R.2 Supplementary material

Vitamin C (µg)	6.6	6.6
Vitamin D (µg)	1.2	1.2
Vitamin E (mg)	0.75	0.75
Vitamin K (µg)	6.6	6.6

RCT 7: sn-2 Palmitate enriched formula vs standard formula

per 100 ml	Standard formula	sn-2 Palmitate enriched formula
Energy (kcal)	70	73
Protein (g)	1.6	1.6
Carbohydrate (g)	7.1	7.1
Lactose (g)	7.1	7.1
Total fat (g)	3.9	4.2
Fatty acid composition (% total fat)		
C8:0 caprylic	1.6	1.5
C10:0 capric	0.5	0.4
C12:0 lauric	10.3	12.4
C14:0 myristic	4.9	4.7
C16:0 palmitic	19.6 (12% in sn-2)	20.1 (50% in sn-2)
C18:0 stearic	3.9	3.1
C18:1 n-9 oleic	41.3	42
C18:2 n-6 linoleic	12.1	13
C18:3 n-3 α-linolenic	1.9	1.6
Other fatty acids	4.0	1.2
Minerals		
Calcium (mg)	54	57
Phosphorus (mg)	32	33
Sodium (mg)	23	23
Potassium (mg)	76	74
Chloride (mg)	45	44
Magnesium (mg)	6.3	6.3
Manganese (µg)	5.1	5.2
Iron (mg)	0.51	0.57
Zinc (mg)	0.45	0.49
Copper (µg)	44	45
Iodine (µg)	10	10
Vitamins		
Vitamin A (µg)	92	95
Thiamine B_1 (µg)	40	40
Riboflavin B_2 (µg)	100	100
Pyridoxine B_6 (µg)	40	40
Vitamin D (µg)	1.1	1.1
Vitamin K (µg)	5	5
Vitamin E (mg)	1.1	1.1

For peer review only - http://bmjopen.bmj.com/site/about/guidelines.xhtml

bmjopen-2019-035968 R.2 Supplementary material

References:

1. Lucas A, Fewtrell MS, Morley R, et al. Randomized trial of nutrient-enriched formula versus standard formula for postdischarge preterm infants. Pediatrics 2001;108(3):703-11. [published Online First: 2001/09/05]
2. Fewtrell MS, Morley R, Abbott RA, et al. Catch-up growth in small-for-gestational-age term infants: a randomized trial. The American journal of clinical nutrition 2001;74(4):516-23. doi: 10.1093/ajcn/74.4.516 [published Online First: 2001/09/22]
3. Morley R, Fewtrell MS, Abbott RA, et al. Neurodevelopment in children born small for gestational age: a randomized trial of nutrient-enriched versus standard formula and comparison with a reference breastfed group. Pediatrics 2004;113(3 Pt 1):515-21. [published Online First: 2004/03/03]
4. Fewtrell MS, Morley R, Abbott RA, et al. Double-Blind, Randomized Trial of Long-Chain Polyunsaturated Fatty Acid Supplementation in Formula Fed to Preterm Infants. Pediatrics 2002;110(1):73-82. doi: 10.1542/peds.110.1.73
5. Fewtrell MS, Abbott RA, Kennedy K, et al. Randomized, double-blind trial of long-chain polyunsaturated fatty acid supplementation with fish oil and borage oil in preterm infants. The Journal of pediatrics 2004;144(4):471-9. doi: 10.1016/j.jpeds.2004.01.034 [published Online First: 2004/04/08]
6. Lucas A, Stafford M, Morley R, et al. Efficacy and safety of long-chain polyunsaturated fatty acid supplementation of infant-formula milk: a randomised trial. Lancet (London, England) 1999;354(9194):1948-54. doi: 10.1016/s0140-6736(99)02314-4 [published Online First: 2000/01/06]
7. Singhal A, Morley R, Cole TJ, et al. Infant nutrition and stereoacuity at age 4-6 y. The American journal of clinical nutrition 2007;85(1):152-9. doi: 10.1093/ajcn/85.1.152 [published Online First: 2007/01/09]
8. Singhal A, Kennedy K, Lanigan J, et al. Dietary Nucleotides and Early Growth in Formula-Fed Infants: A Randomized Controlled Trial. Pediatrics 2010;126(4):e946-e53. doi: 10.1542/peds.2009-2609
9. Singhal A, Macfarlane G, Macfarlane S, et al. Dietary nucleotides and fecal microbiota in formula-fed infants: a randomized controlled trial. The American journal of clinical nutrition 2008;87(6):1785-92. [published Online First: 2008/06/11]
10. Morley R, Abbott R, Fairweather-Tait S, et al. Iron fortified follow on formula from 9 to 18 months improves iron status but not development or growth: a randomised trial. Archives of Disease in Childhood 1999;81(3):247-52. doi: 10.1136/adc.81.3.247
11. Singhal A, Morley R, Abbott R, et al. Clinical safety of iron-fortified formulas. Pediatrics 2000;105(3):E38. doi: 10.1542/peds.105.3.e38 [published Online First: 2000/03/04]
12. Kennedy K, Fewtrell MS, Morley R, et al. Double-blind, randomized trial of a synthetic triacylglycerol in formula-fed term infants: effects on stool biochemistry, stool characteristics, and bone mineralization. The American journal of clinical nutrition 1999;70(5):920-7. doi: 10.1093/ajcn/70.5.920 [published Online First: 1999/10/28]
13. Fewtrell MS, Kennedy K, Murgatroyd PR, et al. Breast-feeding and formula feeding in healthy term infants and bone health at age 10 years. Br J Nutr 2013;110(6):1061-7. doi: 10.1017/s0007114512006149 [published Online First: 2013/02/08]
14. Fellegi IP, Sunter AB. A Theory for Record Linkage. Journal of the American Statistical Association 1969;64(328):1183-210. doi: 10.1080/01621459.1969.10501049

For peer review only - http://bmjopen.bmj.com/site/about/guidelines.xhtml

STANDARD PROTOCOL ITEMS: RECOMMENDATIONS FOR INTERVENTIONAL TRIALS

SPIRIT 2013 Checklist: Recommended items to address in a clinical trial protocol and related documents*

Section/item	Item No	Description	Page
**Administrative information**			
Title	1	Descriptive title identifying the study design, population, interventions, and, if applicable, trial acronym	1
Trial registration	2a	Trial identifier and registry name. If not yet registered, name of intended registry	n/a
	2b	All items from the World Health Organization Trial Registration Data Set	n/a
Protocol version	3	Date and version identifier	all
Funding	4	Sources and types of financial, material, and other support	13
Roles and responsibilities	5a	Names, affiliations, and roles of protocol contributors	1
	5b	Name and contact information for the trial sponsor	1
	5c	Role of study sponsor and funders, if any, in study design; collection, management, analysis, and interpretation of data; writing of the report; and the decision to submit the report for publication, including whether they will have ultimate authority over any of these activities	13
	5d	Composition, roles, and responsibilities of the coordinating centre, steering committee, endpoint adjudication committee, data management team, and other individuals or groups overseeing the trial, if applicable (see Item 21a for data monitoring committee)	13
**Introduction**			
Background and rationale	6a	Description of research question and justification for undertaking the trial, including summary of relevant studies (published and unpublished) examining benefits and harms for each intervention	13
	6b	Explanation for choice of comparators	n/a
Objectives	7	Specific objectives or hypotheses	4-5

For peer review only

Trial design	8	Description of trial design including type of trial (eg, parallel group, crossover, factorial, single group), allocation ratio, and framework (eg, superiority, equivalence, noninferiority, exploratory)	5

Methods: Participants, interventions, and outcomes

Study setting	9	Description of study settings (eg, community clinic, academic hospital) and list of countries where data will be collected. Reference to where list of study sites can be obtained	Supplementary material Table 1
Eligibility criteria	10	Inclusion and exclusion criteria for participants. If applicable, eligibility criteria for study centres and individuals who will perform the interventions (eg, surgeons, psychotherapists)	Supplementary material Table 1
Interventions	11a	Interventions for each group with sufficient detail to allow replication, including how and when they will be administered	Supplementary material Compositions
	11b	Criteria for discontinuing or modifying allocated interventions for a given trial participant (eg, drug dose change in response to harms, participant request, or improving/worsening disease)	n/a
	11c	Strategies to improve adherence to intervention protocols, and any procedures for monitoring adherence (eg, drug tablet return, laboratory tests)	Supplementary material
	11d	Relevant concomitant care and interventions that are permitted or prohibited during the trial	n/a

For peer review only - http://bmjopen.bmj.com/site/about/guidelines.xhtml

Outcomes	12	Primary, secondary, and other outcomes, including the specific measurement variable (eg, systolic blood pressure), analysis metric (eg, change from baseline, final value, time to event), method of aggregation (eg, median, proportion), and time point for each outcome. Explanation of the clinical relevance of chosen efficacy and harm outcomes is strongly recommended	6
Participant timeline	13	Time schedule of enrolment, interventions (including any run-ins and washouts), assessments, and visits for participants. A schematic diagram is highly recommended (see Figure)	Figure 1
Sample size	14	Estimated number of participants needed to achieve study objectives and how it was determined, including clinical and statistical assumptions supporting any sample size calculations	10
Recruitment	15	Strategies for achieving adequate participant enrolment to reach target sample size	Supplementary material

Methods: Assignment of interventions (for controlled trials)

Allocation:

Sequence generation	16a	Method of generating the allocation sequence (eg, computer-generated random numbers), and list of any factors for stratification. To reduce predictability of a random sequence, details of any planned restriction (eg, blocking) should be provided in a separate document that is unavailable to those who enrol participants or assign interventions	n/a
Allocation concealment mechanism	16b	Mechanism of implementing the allocation sequence (eg, central telephone; sequentially numbered, opaque, sealed envelopes), describing any steps to conceal the sequence until interventions are assigned	n/a
Implementation	16c	Who will generate the allocation sequence, who will enrol participants, and who will assign participants to interventions	n/a
Blinding (masking)	17a	Who will be blinded after assignment to interventions (eg, trial participants, care providers, outcome assessors, data analysts), and how	n/a

	17b	If blinded, circumstances under which unblinding is permissible, and procedure for revealing a participant's allocated intervention during the trial	n/a
**Methods: Data collection, management, and analysis**			
Data collection methods	18a	Plans for assessment and collection of outcome, baseline, and other trial data, including any related processes to promote data quality (eg, duplicate measurements, training of assessors) and a description of study instruments (eg, questionnaires, laboratory tests) along with their reliability and validity, if known. Reference to where data collection forms can be found, if not in the protocol	5
	18b	Plans to promote participant retention and complete follow-up, including list of any outcome data to be collected for participants who discontinue or deviate from intervention protocols	5
Data management	19	Plans for data entry, coding, security, and storage, including any related processes to promote data quality (eg, double data entry; range checks for data values). Reference to where details of data management procedures can be found, if not in the protocol	Supplementary material
Statistical methods	20a	Statistical methods for analysing primary and secondary outcomes. Reference to where other details of the statistical analysis plan can be found, if not in the protocol	8
	20b	Methods for any additional analyses (eg, subgroup and adjusted analyses)	9
	20c	Definition of analysis population relating to protocol non-adherence (eg, as randomised analysis), and any statistical methods to handle missing data (eg, multiple imputation)	10
**Methods: Monitoring**			
Data monitoring	21a	Composition of data monitoring committee (DMC); summary of its role and reporting structure; statement of whether it is independent from the sponsor and competing interests; and reference to where further details about its charter can be found, if not in the protocol. Alternatively, an explanation of why a DMC is not needed	n/a
	21b	Description of any interim analyses and stopping guidelines, including who will have access to these interim results and make the final decision to terminate the trial	n/a

For peer review only - http://bmjopen.bmj.com/site/about/guidelines.xhtml

Harms	22	Plans for collecting, assessing, reporting, and managing solicited and spontaneously reported adverse events and other unintended effects of trial interventions or trial conduct	n/a
Auditing	23	Frequency and procedures for auditing trial conduct, if any, and whether the process will be independent from investigators and the sponsor	n/a
**Ethics and dissemination**			
Research ethics approval	24	Plans for seeking research ethics committee/institutional review board (REC/IRB) approval	12
Protocol amendments	25	Plans for communicating important protocol modifications (eg, changes to eligibility criteria, outcomes, analyses) to relevant parties (eg, investigators, REC/IRBs, trial participants, trial registries, journals, regulators)	12
Consent or assent	26a	Who will obtain informed consent or assent from potential trial participants or authorised surrogates, and how (see Item 32)	12
	26b	Additional consent provisions for collection and use of participant data and biological specimens in ancillary studies, if applicable	12
Confidentiality	27	How personal information about potential and enrolled participants will be collected, shared, and maintained in order to protect confidentiality before, during, and after the trial	12-13
Declaration of interests	28	Financial and other competing interests for principal investigators for the overall trial and each study site	14
Access to data	29	Statement of who will have access to the final trial dataset, and disclosure of contractual agreements that limit such access for investigators	14
Ancillary and post-trial care	30	Provisions, if any, for ancillary and post-trial care, and for compensation to those who suffer harm from trial participation	n/a
Dissemination policy	31a	Plans for investigators and sponsor to communicate trial results to participants, healthcare professionals, the public, and other relevant groups (eg, via publication, reporting in results databases, or other data sharing arrangements), including any publication restrictions	13
	31b	Authorship eligibility guidelines and any intended use of professional writers	n/a

For peer review only

	31c	Plans, if any, for granting public access to the full protocol, participant-level dataset, and statistical code	14
**Appendices**			
Informed consent materials	32	Model consent form and other related documentation given to participants and authorised surrogates	n/a
Biological specimens	33	Plans for collection, laboratory evaluation, and storage of biological specimens for genetic or molecular analysis in the current trial and for future use in ancillary studies, if applicable	n/a

*It is strongly recommended that this checklist be read in conjunction with the SPIRIT 2013 Explanation & Elaboration for important clarification on the items. Amendments to the protocol should be tracked and dated. The SPIRIT checklist is copyrighted by the SPIRIT Group under the Creative Commons "Attribution-NonCommercial-NoDerivs 3.0 Unported" license.

For peer review only
